# Cyanidin‐3‐O‐glucoside ameliorates hydrogen peroxide‐induced oxidative stress by regulating HMGCR‐mediated cholesterol anabolism in HEK‐293T cells

**DOI:** 10.1002/fsn3.4231

**Published:** 2024-07-01

**Authors:** Di Liu, Hanxue Zhang, Yu Dai, Jie Sun, Hongyu Sun, Zixiang Yu, Fanli Kong, Xianmin Feng

**Affiliations:** ^1^ College of Basic Medicine Jilin Medical University Jilin China; ^2^ College of Medical Technology Beihua University Jilin China; ^3^ Department of Clinical Laboratory Suzhou Hospital of Traditional Chinese Medicine Suzhou China

**Keywords:** anthocyanidins, cholesterol, cyanidin‐3‐O‐glucoside, HMGCR, oxidative stress, ROS

## Abstract

Cyanidin‐3‐O‐glucoside (C3G), as a typical anthocyanin, exhibits excellent antioxidant effects. This study aimed to demonstrate the role and mechanism of C3G in regulating 3‐hydroxy‐3‐methylglutaryl‐coenzyme A reductase (HMGCR)‐mediated cholesterol anabolism on H_2_O_2_‐induced oxidative stress in HEK‐293T cells. Firstly, the inhibitory effect of C3G on oxidative stress was confirmed by CCK‐8, ROS, and mitochondrial membrane potential (MMP) experiments. Then, proteomics was used to investigate and screen differentially expressed proteins in inhibiting cellular oxidative stress by C3G. HMGCR was screened as a key differentially expressed protein by proteomic analysis. The results verified that C3G could reduce cholesterol levels by inhibiting sterol regulatory element‐binding protein (SREBP2)/HMGCR pathway, increasing ATP, and reducing acetyl‐CoA. Finally, HMGCR had been shown to positively increase ROS accumulation and decrease MMP, which were reversed by intervention of C3G through a series of knockdown and overexpression experiments. In conclusion, the results demonstrated that C3G could inhibit the disorder of cholesterol synthesis in oxidative stress cells by regulating the ROS/SREBP2/HMGCR pathway.

## Graphical abstract

HMGCR is a target protein screened by proteomic analysis, it mediated cholesterol synthesis plays a central role in the inhibition of oxidative stress progression by C3G. C3G reduces cholesterol synthesis by inhibiting sterol regulatory element‐binding protein (SREBP2)/3‐hydroxy‐3‐methylglutaryl‐coenzyme A reductase (HMGCR) pathway, increasing ATP and reducing acetyl‐CoA. C3G reverses the positive effect of HMGCR on ROS accumulation and decreases mitochondrial membrane potential.

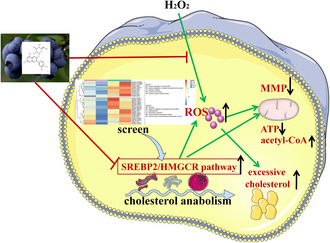

## 1 INTRODUCTION

Oxidative stress occurs when there is an imbalance in excessive oxidants (including ROS and RNS) and antioxidants, always accompanied by dysfunction of redox circuits and macromolecular damage (van der Pol et al., 2019; Zarneshan et al., 2022). ROS are the products of cellular aerobic metabolism. As the second messenger of cell signal transduction, ROS in low concentrations are involved in the regulation of physiological processes (Matés et al., 2008). ROS‐induced lipid peroxides are key mediators of aging, inflammation, metabolic disorders, and chronic diseases (Gaschler & Stockwell, 2017). As an important component of lipids, cholesterol is involved in many physiological processes such as biofilm flow, transmembrane transport, signal transduction, and functional regulation of membrane proteins (Frey et al., 2018). Homeostasis of cellular cholesterol is the key to regulate the accurate renewal of lipids and maintain the stability of cell membrane and the normal physiological function of cells. However, cholesterol is highly susceptible to ROS‐induced peroxidation, resulting in disrupted cholesterol metabolism.

Anthocyanidins are a well‐known group of dietary antioxidants. *Cyanidin‐3‐O‐glucoside* (C3G) is the most abundant anthocyanin glucoside in nature, mainly found in grapes, blueberries, black rice, black beans, purple potato, purple cabbage, and other plant foods (Kamiloglu et al., 2015; Lila et al., 2016). Studies have shown that a diet rich in anthocyanins can prevent a variety of free radical‐mediated human sub‐health and chronic diseases, which is attributed to the antioxidant and anti‐inflammatory activities of anthocyanins (Li, Zhao, et al., 2021). The antioxidative capacity of anthocyanin is not only reflected in the excellent scavenging activity of free radicals but also related to the activation and improvement of endogenous antioxidant defense and biological metabolic system. Anthocyanins can effectively reduce the formation of cholesterol oxides by inhibiting lipid peroxidation (Ferreira et al., 2022), indirectly activate specific detoxification enzymes, and reduce oxidative stress caused by ROS (Ajit et al., 2016; Ferrari et al., 2017). In recent years, the antioxidant research of anthocyanin related to cellular metabolism disorders has also received attention. Studies have shown that C3G can effectively reduce the release of lipid peroxidation products, such as 4‐hydroxynonenal and malondialdehyde, inhibit lipid peroxidation through targeting ROS, and thus alleviate metabolic dysfunction in cells (Ferrari et al., 2017; Sun et al., 2016). Although the anti‐lipid oxidation effect of anthocyanins has been well established, direct evidence about effect of anthocyanins on the disturbance of cholesterol metabolism under oxidative stress is lacking.

Proteomics have made it possible to understand complex biological systems and determine the relationships, functions, and interactions between proteins (Li et al., 2018). This approach can be used to precisely evaluate the changes in protein expression under dietary intervention, which is conducive to obtaining the potential molecular mechanism and action targets of dietary active ingredients. Isobaric labeling‐based proteomics were used to compare antifibrotic activities of scutellariae radix extracts and flavonoids (Zhou et al., 2022). Tandem mass tag (TMT)‐based proteomics and bioinformatics analyses had been used to reveal that apoptosis‐related signaling pathways played pivotal roles in the action of C3G‐mediated protection against heterocyclic aromatic amines induced cytotoxicity (Zhao et al., 2021). It was found that flavonoids and C3G could improve diabetic nephropathy by regulating AGE‐RAGE pathway and amino acid metabolism by using the label‐free quantification proteomics (Li et al., 2022; Li, Yang, et al., 2021).

In this study, a series of highly sensitive quantitative approaches including TMT labeling, LC–MS/MS‐based quantitative proteomics and advanced bioinformatics analysis were organically combined to explore the changes of global proteome during the intervention of C3G in oxidative stress. Based on the data analysis, the molecular mechanism of C3G regulating intracellular cholesterol metabolism to inhibit oxidative stress was further investigated through functional verification and gene intervention. It will provide a new perspective for the study on antioxidant of active ingredients in foods represented by anthocyanins.

## 2 MATERIALS AND METHODS

### 2.1 Reagents and chemicals

*Cyanidin‐3‐O‐glucoside* (C3G), molecular formula C_21_H_21_ClO_11_ and molecular weight 449.38, was purchased from Shanghai Yuanye Bio‐Technology Co., Ltd. (>95% purity; Shanghai, China). H_2_O_2_, protease inhibitor cocktail, TMT kit, acetonitrile (ACN), and pure water were obtained from Thermo Fisher Scientific (Waltham, MA, USA). Formic acid (FA) was obtained from Fluka (Buchs, Switzerland). Dulbecco's Modified Eagle's Medium (DMEM) and Penicillin–Streptomycin Solution (PSS) were purchased from Gibco (Grand Island, NY, USA). Fetal Bovine Serum (FBS) was obtained from Biological Industries (BI, Israel). Sequencing Grade Modified Trypsin was purchased from Promega (Fitchburg, WI, USA). The cell counting kit‐8 (CCK‐8) assay kit was from Bioss (Beijing, China). Rabbit antibodies against GAPDH, HMGCR, SREBP2, goat anti‐mouse, and goat anti‐rabbit antibodies were from Abcam (Cambrige, UK). Polyvinylidene difluoride (PVDF) membranes were obtained from Millipore Corporation (Billerica, MA, USA). HMGCR siRNA was from Guangzhou RiboBio Co., Ltd. (Guangzhou, China). Assay kits for detection of ROS, Mitochondria Membrane Potential (MMP), ATP, RNA isolation (Spin Column), QuantiTect Reverse Transcription, total protein (based BCA method), and enhanced ECL reagent were obtained from Beyotime Biotechnology (Shanghai, China). The assay kit for the detection of Acetyl‐CoA was obtained from Shanghai Enzyme‐linked Biotechnology Co., Ltd. (Shanghai, China). The assay kit for detection of cholesterol was obtained from Applygen Technologies Inc. (Beijing, China). SYBR qPCR SuperMix Plus was obtained from Novoprotein Scientific Inc (Shanghai, China). Trifluoroacetic acid (TFA), iodoacetamide (IAA), dithiothreitol (DTT), urea, EDTA, and TEAB were purchased from Sigma (St. Louis, MO, USA). Lipofectamine 3000 reagent was purchased from Invitrogen (Carlsbad, CA, USA). The pcDNA3.1(+) vector of full‐length HMGCR (Cat No. PPL00622‐2a) and empty vector plasmid were obtained from the Public Protein/Plasmid Library (Biogot Tech, Nanjing, China). HMGCR siRNA reagents and negative control siRNA were obtained from RiboBio (Guangzhou, China).

### 2.2 Cell culture and treatment

HEK‐293T cells (purchased from the Center of Excellence in Molecular and Cellular Science, Chinese Academy of Sciences, Shanghai, China) were cultured in DMEM supplemented with 10% FBS and 1% PSS, and incubated in a humidified incubator containing 5% CO_2_ at 37°C. Cells were seeded in 96‐well or six‐well plates and incubated for 24 h. The cells were pretreated with C3G (20 and 40 μmol/L) for 12 h before being exposed to 600 μmol/L of H_2_O_2_ for 6 h.

### 2.3 CCK‐8 assay

Cell viability, protective effect, and toxic effect were evaluated using the CCK‐8 assay. Briefly, HEK‐293T cells were collected and seeded at a density of 8 × 10^3^ cells/well in 96‐well plates. After various treatments, cells were incubated with 10 μL of CCK‐8 solution for 1 h following the manufacturer's specifications. The data was assessed by measuring the optical density (OD) at 450 nm using a Multimode Plate Reader (Molecular Devices, USA). The results were expressed as a percentage of the corresponding values in the control group.

### 2.4 Measurement of intracellular ROS

The production of intracellular ROS was tested using DCFH‐DA as the tag. In short, HEK‐293T cells were cultivated at a density of 8 × 10^5^ cells/well in six‐well plates. After treatment, cells were incubated in serum‐free medium supplemented with 10 μmol/L of DCFH‐DA for 30 min in the dark. The cells were observed using a laser scanning confocal microscope (Olympus, Japan). ImageJ software (U.S. National Institutes of Health, Bethesda, MD, USA) was used to quantify the intensity of green fluorescence.

### 2.5 Measurement of mitochondrial membrane potential (MMP)

JC‐1 dye was used for evaluating the MMP of HEK‐293T cells. In brief, the cells were cultured in six‐well plates. After treatment, cells were incubated with serum‐free medium supplemented with 5 μmol/L of JC‐1 dye for 20 min in the dark. The cells were observed using a fluorescence microscope (Olympus, Japan). ImageJ software was used to quantify the intensity of red and green fluorescence.

### 2.6 Detections of Acetyl‐CoA, ATP, and cholesterol

For each time point, Acetyl‐CoA, ATP level, and cholesterol were measured using corresponding assay kits. Bioluminescence was detected on a Multimode Plate Reade (Molecular Devices, USA), and further normalized by protein quantification based on BCA assay.

### 2.7 Protein preparation

Cells were sonicated three times on ice using a high‐intensity ultrasonic processor (Scientz, China) in lysis buffer (8 M urea, 1% Protease inhibitor cocktail, and 2 mM EDTA). The supernatant was collected after centrifuging under 12,000 *g* at 4°C for 10 min. The protein concentration was determined with BCA kit. For digestion, the protein solution was reduced with 5 mM DTT under conditions of 56°C and 30 min. Then it was alkylated with 11 mM IAA under the condition of 25°C and 15 min in the dark. The urea concentration of the sample was diluted to less than 2 M with 100 mM TEAB. Enzymolysis was performed at 1:50 and 1:100 trypsin‐to‐protein mass ratio for 12 and 4 h, respectively.

### 2.8 TMT labeling

The enzymatic peptides were desalted by Strata X C18 SPE column (Phenomenex, Torrance, CA) and freeze‐dried under vacuum. Then they were dissolved in 0.5 M TEAB and labeled according to the instructions of the TMT kit. Briefly, the acetonitrile‐dissolved labeling reagent was mixed with the peptide and incubated at room temperature for 2 h, followed by desalting and vacuum freeze‐drying.

### 2.9 Bioinformatics analysis

Hierarchical clustering analysis based on differentially expressed proteins (DEPs) was carried out using Cluster3.0 software. A heat map was constructed to better visualize and identify the between groups. For the convenience of gene annotation, corresponding ensemble gene IDs of the DEPs were used for further bioinformatics analysis.

Gene Ontology (GO) annotation proteome was derived from the UniProt‐GOA database (http://www.ebi.ac.uk/GOA/). Proteins were classified by GO annotation into three categories: biological process, cellular compartment, and molecular function. For each category, a two‐tailed Fisher's exact test was employed to test the enrichment of the differentially expressed protein against all identified proteins. The GO with a corrected *p*‐value <.05 is considered significant. Protein–protein interaction (PPI) analysis was performed using the STRING database (http://string‐db.org/).

### 2.10 HMGCR overexpression and knockdown

Cells were cultivated in six‐well plates for 24 h and transfected with HMGCR‐specific siRNA (5 nmol, target sequence of siRNA1: GACAGAATCTACACTCTCA, siRNA2: GTTCCAGAATTTACGTCAA, siRNA3: GGATGAACATGATTTCAAA) or siRNA‐NC using the Lipofectamine 3000 reagent. The overexpression of HMGCR was performed via transfection of human pcDNA3.1(+)‐HMGCR‐3*Flag overexpression plasmid. After transfection for 24 h, the cells were exposed to H_2_O_2_ after incubation with or without pretreatment of C3G.

### 2.11 Western blotting

Briefly, protein fractions (30 μg) were separated by 8% SDS‐PAGE gel electrophoresis and transferred onto PVDF membranes in Tris‐glycine buffer at 200 mA. The PVDF membranes were blocked with Tris buffer supplemented with 0.1% (v/v) Tween 20 (TBST) and 5% (w/v) skim milk for 2 h at room temperature. Subsequently, membranes were incubated with HMGCR (1:1000), SREBP2 (1:1000), and GAPDH (1:3000) antibodies overnight at 4°C. After washing three times with TBST, the membranes were incubated with secondary antibodies for 1 h at room temperature. Finally, the protein bands were visualized using a chemiluminescence imaging analyzer (Tanon, Shanghai, China) after exposure to ECL reagent. The protein expression signals were analyzed by Image J and normalized to GAPDH.

### 2.12 qRT‐PCR

Total RNA samples from the cultured HEK‐293T cells described above were separated using RNA Isolation Kit. Total RNA (1 μg) was reverse‐transcribed to cDNA using a reverse transcriptase kit. A series of HMGCR, SREBP2, and GAPDH primers were selected by Primer Premier 5.0 software. The sequences of the primers were:

GAPDH (forward primer 5′‐GGTGAAGGTCGGAGTCAACG‐3′, reverse primer 5′‐CAAAGTTGTCATGGATGHACC‐3′),
HMGCR (forward primer 5′‐CCAGAGCAAGCACATTAGC‐3′, reverse primer 5′‐CAGCCAAAGCAGCACATA‐3′),
SREBP2 (forward primer 5′‐GAGGCTGAGTTGCTGTAG‐3′, reverse primer 5′‐TGTGGAGGTAGGAGATGG‐3′).

The levels of gene expression were evaluated by ABI 7500 fast real‐time PCR system (Applied Biosystems, Foster City, CA, USA) with SYBR qPCR SuperMix Plus. The expression level of the target gene relative to GAPDH was calculated by the comparative *C*_t_ method formula: $2^{-∆∆C_{t}}$.

### 2.13 Statistical analysis

Results were presented as means ± SD from experiments in triplicate. The average value of all experiments was standardized to the control group and used for statistical analysis. Statistical comparisons among different groups were determined by one‐way ANOVA test followed by the least significant difference (LSD) test using SPSS software 19.0. Differences were deemed to be statistically significant when *p <* .05.

## 3 RESULTS

### 3.1 C3G attenuated H_2_O_2_ ‐induced cell damage

**FIGURE 1:**
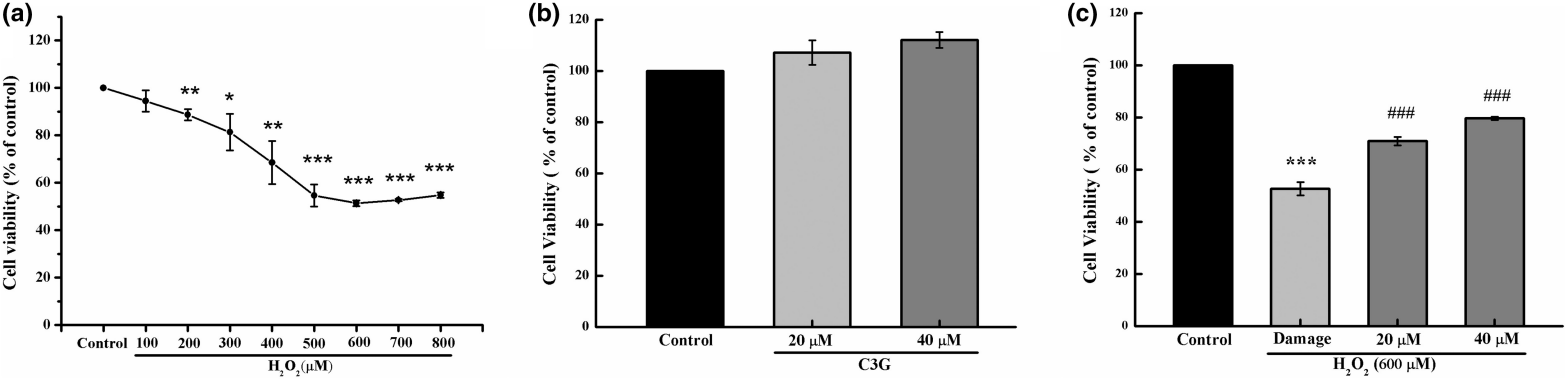
Protective effect of C3G on H_2_O_2_ induced oxidative stress in HEK‐293T cells. (a) Cells were treated with different concentrations of H_2_O_2_ for 6 h. (b) Cells were treated with various concentrations of C3G (20 and 40 μM) for 12 h. (c) Cells were pretreated with various concentrations of C3G (20 and 40 μM) for 12 h followed by treatment of H_2_O_2_ (600 μM) for 6 h. Cell viability was detected by CCK‐8 assay. The results were represented as means ± SD from experiments in triplicate. *p < .05, **p < .01, ***p < .001 versus control group; ^###^ *p <* .001 versus damage group.

As presented in Figure 1a, the cell viability decreased in a concentration‐dependent manner from 100 to 600 μM and tended to flatten out after greater than 600 μM. The cell viability decreased to 51.40% when cells were treated with 600 μM H_2_O_2_. There was no significant change in cell viability after incubation with C3G at the concentrations of 20 and 40 μM (Figure 1b). After excluding the possibility of proliferation and cytotoxicity, the protective effect of C3G against oxidative stress in HEK‐293T cells were determined by CCK‐8 assay. As presented in Figure 1c, the cell viability decreased to approximately 52.62% after exposure to H_2_O_2_. Compared to the damage group, the cell viability elevated to 70.92% (*p <* .001) and 79.62% (*p <* .001) when pretreated with 20 and 40 μM of C3G under exposure with H_2_O_2_.

### 3.2 C3G alleviated the H_2_O_2_ ‐induced ROS generation and MMP reduction

**FIGURE 2:**
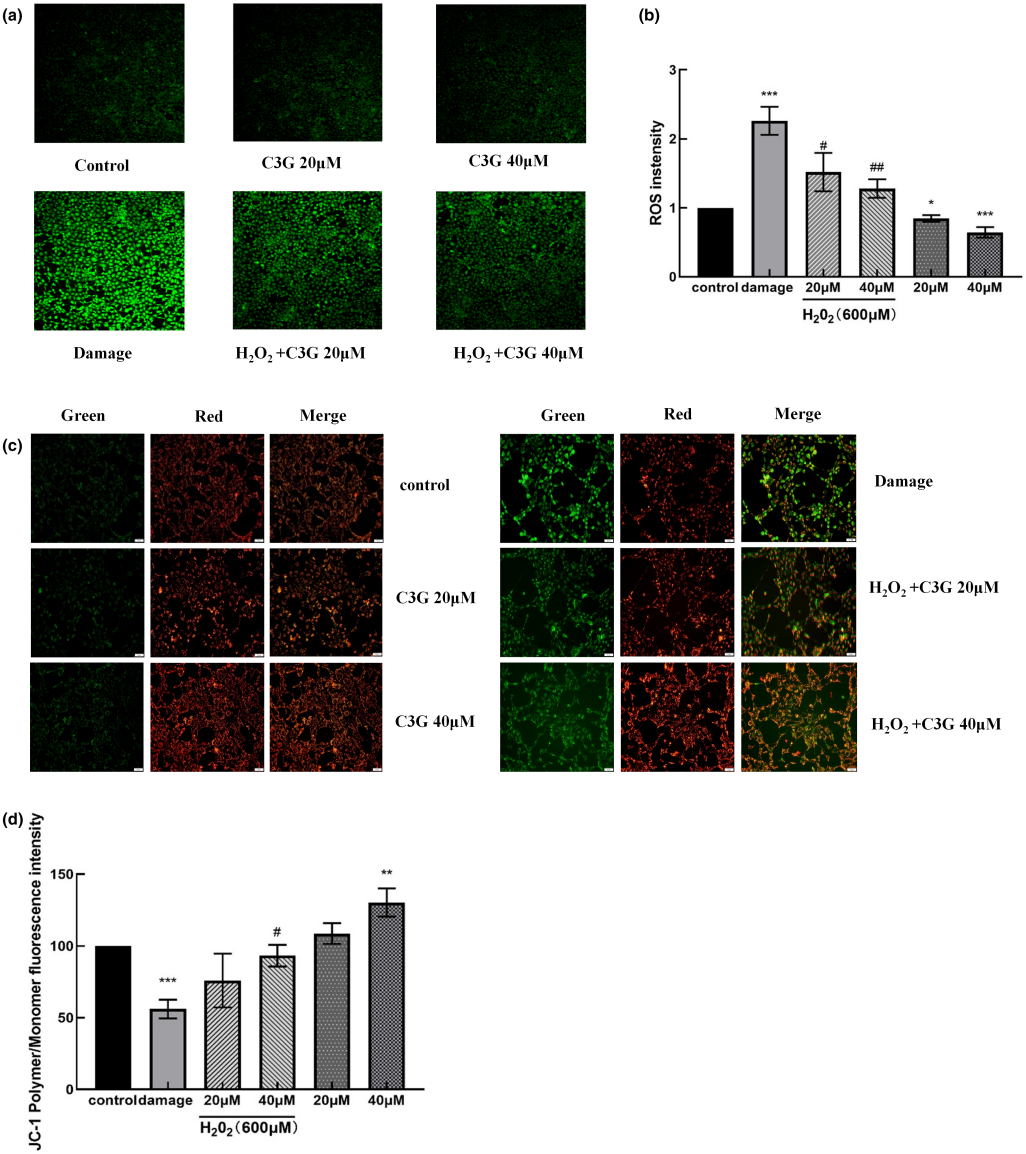
Effects of C3G on intracellular ROS production and MMP changes in HEK‐293T cells. Cells were pretreated with various concentrations of C3G (20 and 40 μM) for 12 h followed by treatment with or without H_2_O_2_ (600 μM) for 6 h. (a) Images of DCFH‐DA fluorescence staining were observed by a laser scanning confocal microscope. (b) Intracellular ROS levels were analyzed by ImageJ. (c) Images of JC‐1 fluorescence staining were observed by a fluorescence microscope. (d) Quantitative analysis of the MMP was analyzed by ImageJ and represented by relative red/green ratio. The results were represented as means ± SD from experiments in triplicate. *p < .05, **p < .01, ***p < .001, versus control group; ^#^ *p <* .05, ^##^ *p <* .01 versus damage group.

To further clarify the effect of C3G on H_2_O_2_‐induced oxidative stress, the level of intracellular ROS was examined by DCFH‐DA fluorescence assay. As shown in Figure 2, the enhanced green fluorescence and elevated ROS levels were observed in H_2_O_2_ treatment cells, while the administration of C3G reduced the H_2_O_2_‐induced intracellular ROS generation (Figure 2a,b).

Mitochondrial membrane potential (MMP) was detected to assess whether mitochondrial protection is involved in the inhibitory effect of C3G on ROS‐induced oxidative stress. As shown in Figure 2c,d, the enhanced green fluorescence was observed after exposure to H_2_O_2_, as well as depressed red/green fluorescence ratio, which suggested that MMP decreased in the damage group. In contrast, pretreatment of 40 μM C3G significantly elevated the red/green fluorescence ratio (*p* < .05), which is a sign of an improved MMP.

### 3.3 Analysis and identification of differential protein expression

**FIGURE 3:**
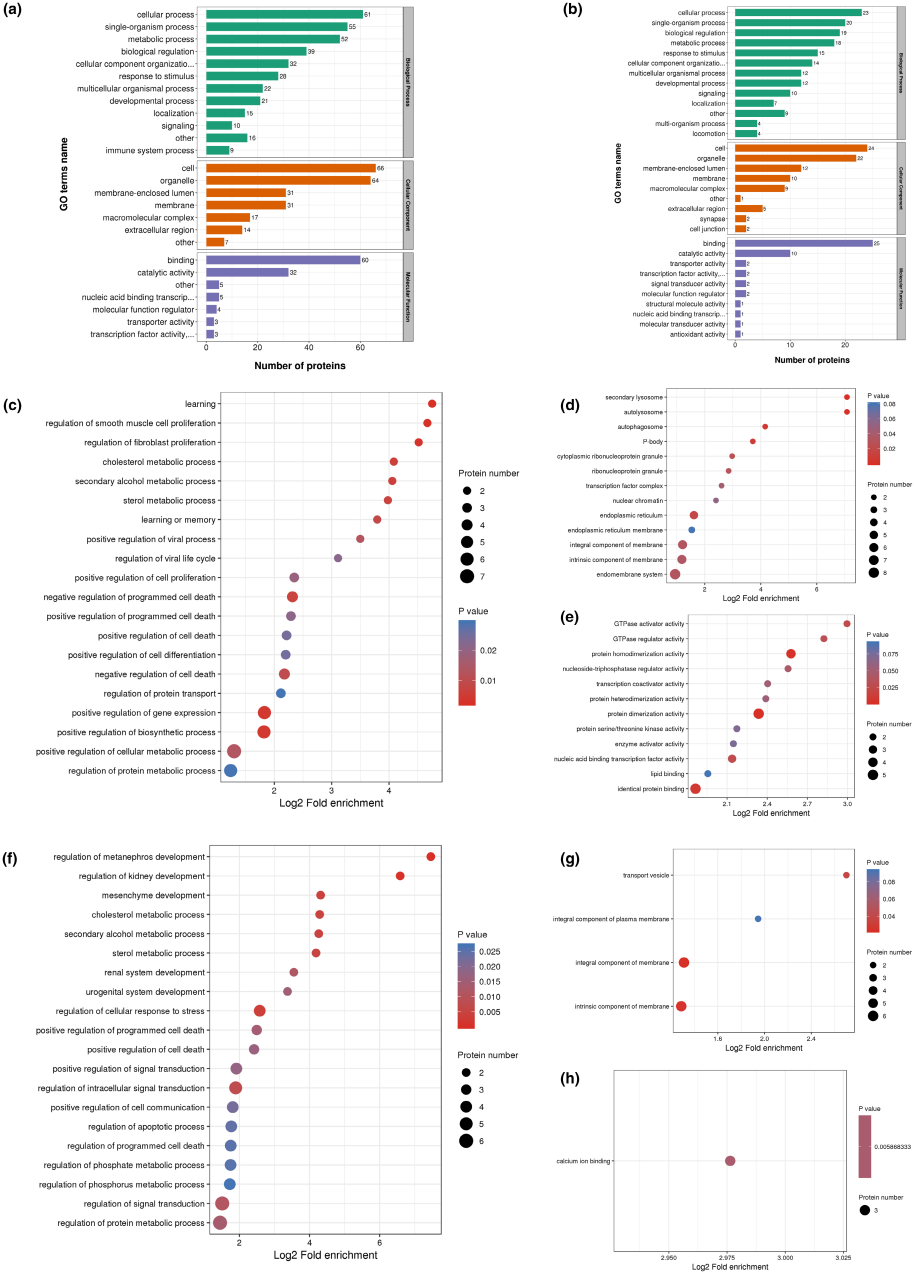
Proteomic analysis of HEK‐293T oxygen‐damaged cells pretreated with C3G. Cells were pretreated with various concentrations of C3G (40 μM) for 12 h followed by treatment of H_2_O_2_ (600 μM) for 6 h. (a) Statistical distribution chart of differentially expressed proteins (DEPs) under each GO category (2nd Level) (S/D). (b) Statistical distribution chart of DEPs under each GO category (2nd Level) (C/S). (c–h) GO enrichment bubble plot of DEPs in three categories. Upregulated DEPs of S/D enriched in Biological Process (c), Cellular Component (d), Molecular Function (e); Downregulated DEPs of C/S enriched in Biological Process (f), Cellular Component (g), Molecular Function (h). The results were analyzed from experiments in triplicate. S/D: damage group versus control group, C/S: C3G intervention group for oxidative damage versus damage group.

A total of 6411 proteins were quantified by proteomic analysis. When *p*‐value <.05, the change of differential expression level was more than 1.2‐fold as the significantly upregulated change threshold, and less than 0.83‐fold as the significantly downregulated change threshold. Finally, 15 proteins were upregulated, 55 proteins were downregulated in damage group (S) compared with the control group (D). And 12 proteins were upregulated, 16 proteins were downregulated in C3G protective group (C) compared with the damage group (S). In order to clarify the biological correlation of the identified proteins, Gene Ontology (GO) is used to classify the molecular functions and biological processes (biological processes‐BP, cell components‐CC, molecular functions‐MF) of differentially expressed proteins (DEPs). As shown in Figure 3a,b, in biological processes, the identified DEPs of S versus D were annotated as metabolic process (52), response to stimulus (28), signaling (10), etc. In cells the DEPs were mainly annotated to membrane‐related components. In molecular functions, the identified DEPs were annotated as binding (60), catalytic activity (32), transporter and transcription activities (6), etc. The annotated classification of DEPs among S versus D was like that of C versus S in biological processes and cell components. However, many differences were shown in the molecular function, such as signal transducer activity, structural molecule activity, and antioxidant activity. The information on annotation and quantification for DEPs were exhibited in Tables S1 and S2.

To reveal the nature of the DEPs in damaged cells and C3G pretreatment cells, GO enrichment‐based clustering was carried out. The GO enrichment analysis demonstrated that the upregulated proteins in the S/D compared group and downregulated proteins in the C/S compared group significantly accumulated in the CC, MF, and BP categories, being associated with terms including integral component of membrane, intrinsic component of membrane, calcium ion binding, cholesterol metabolic process, sterol metabolic process, regulation of cellular response to stress, positive regulation of cell death (Figure 3c–h). The downregulated proteins in S/D group but unchanged in C/S group significantly accumulated in the terms including regulation of superoxide metabolic process, positive regulation of oxidoreductase activity, sterol biosynthetic process, execution phase of apoptosis, basement membrane, extracellular matrix component, histone acetyltransferase complex (Figure S1A–C). The upregulated proteins in C/S group but unchanged in S/D group significantly accumulated in the terms including positive regulations of programmed cell death and apoptotic process, ATPase activity (Figure S1D–F).

**FIGURE 4:**
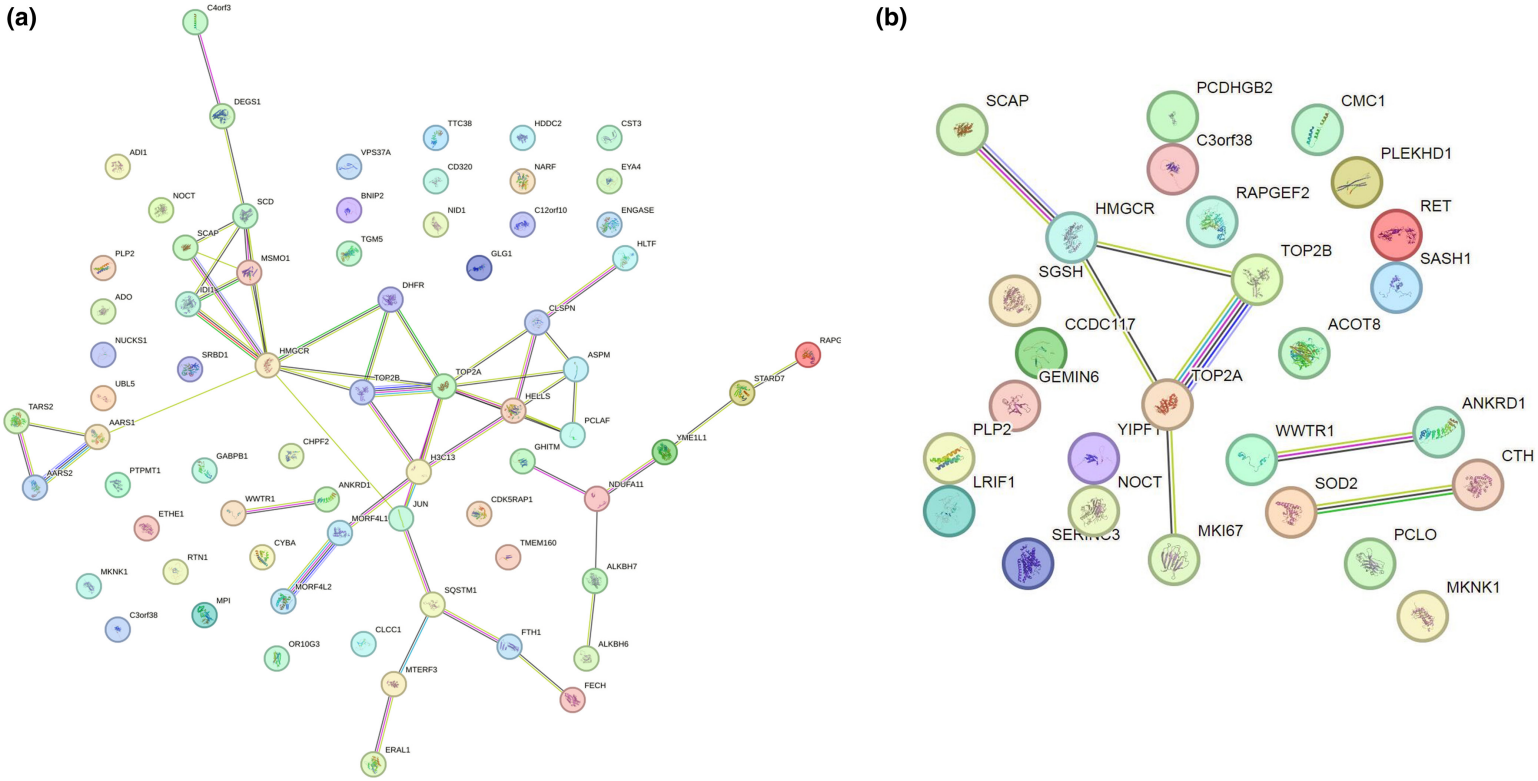
The protein–protein interaction networks of DEPs. Network was constructed by the STRING database. (a) S/D: damage group versus control group. (b) C/S: C3G intervention group for oxidative damage versus damage group.

To gain novel insight into interaction networks involved in oxidative stress and C3G intervention, the PPI network was searched using the STRING online database (Figure 4). Thirty‐five DAPs in the S/D group were involved protein–protein interaction, 8 of which were upregulated and the rest downregulated (Figure 4a, Table S1). Some proteins that were significantly altered by H_2_O_2_ interact with each other, such as HMGCR–SCAP–TOP2B–TOP2A–MORF4L1–MORF4L2, AARS1–AARS2–TARS2, WWTR1–ANKRD1, NDUFA11–GHITM–YME1L1. These proteins have important functions in sterol‐sensing and biosynthesis, replication, transcription and recombination of DNA, cell proliferation and apoptosis, protein biosynthesis, and growth‐related proteins. Nine DAPs in the C/S group were involved in protein–protein interaction, of which five were upregulated and the rest downregulated (Figure 4b, Table S2). The central proteins including HMGCR–SCAP–TOP2B–TOP2A–MK167, WWTR1–ANKRD1, and SOD2–CTH were closely interacted with each other. These proteins have important functions in sterol‐sensing and biosynthesis, replication, transcription and recombination of DNA, cell proliferation and apoptosis, and anti‐oxidative stress.

Based on the GO analysis, the common DEPs in the two comparison groups were highlighted, especially those that were expressed opposite between the two comparison groups, which were listed in Table 1. Among these proteins, HMGCR was upregulated to 1.545‐fold (*p* = .0252) in the S group compared with the D group, but it was downregulated to 0.735‐fold (*p* = .0398) in the C compared with the S group. HMGCR is of great interest because it is a key regulatory enzyme essential for cellular cholesterol homeostasis, and related to lipid oxidation as well as ROS‐induced membrane damage. However, its role in resisting oxidative stress remains unknown. Thus, the results of proteomics and the regulatory effect of C3G on cholesterol anabolism were first determined by the following experiments.

**TABLE 1:** DEPs that expressed opposite in two comparison groups.^a^

Protein accession	Protein description	Gene name	S/D ratio	Regulated type	S/D	C/S ratio	Regulated type	C/S	Subcellular localization
					*p* Value			*p* Value	
P04035	3‐hydroxy‐3‐methylglutaryl‐coenzyme A reductase OS = Homo sapiens OX = 9606 GN = HMGCR	HMGCR	1.545	Up	.0252	0.735	Down	.0398	Plasma membrane
Q12770	Sterol regulatory element‐binding protein cleavage‐activating protein OS = Homo sapiens OX = 9606 GN = SCAP	SCAP	1.292	Up	.0044	0.588	Down	.0031	Plasma membrane
Q9GZV5	WW domain‐containing transcription regulator protein 1 OS = Homo sapiens OX = 9606 GN = WWTR1	WWTR1	1.39	Up	.0029	0.786	Down	.0437	Nucleus
Q9BUB5	MAP kinase‐interacting serine/threonine‐protein kinase 1 OS = Homo sapiens OX = 9606 GN = MKNK1	MKNK1	1.275	Up	.0303	0.83	Down	.0063	Nucleus
Q02880	DNA topoisomerase 2‐beta OS = Homo sapiens OX = 9606 GN = TOP2B	TOP2B	0.829	Down	.0000	1.241	Up	.0000	Nucleus
P11388	DNA topoisomerase 2‐alpha OS = Homo sapiens OX = 9606 GN = TOP2A	TOP2A	0.781	Down	.0013	1.262	Up	.0000	Nucleus
Q5JPI3	Uncharacterized protein C3orf38 OS = Homo sapiens OX = 9606 GN = C3orf38	C3orf38	0.756	Down	.0002	1.276	Up	.0001	Cytoplasm
Q04941	Proteolipid protein 2 OS = Homo sapiens OX = 9606 GN = PLP2	PLP2	0.832	Down	.0252	1.204	Up	.0181	Plasma membrane
Q6P2S7	Putative tetratricopeptide repeat protein 41 OS = Homo sapiens OX = 9606 GN = TTC41P	TTC41P	0.827	Down	.0184	1.246	Up	.0099	Nucleus
Q9Y4G8	Rap guanine nucleotide exchange factor 2 OS = Homo sapiens OX = 9606 GN = RAPGEF2	RAPGEF2	0.758	Down	.0000	1.4	Up	.0001	Cytoplasm
Q9UK39	Nocturnin OS = Homo sapiens OX = 9606 GN = NOCT	NOCT	0.749	Down	.0397	1.346	Up	.0389	Mitochondria
15327	Ankyrin repeat domain‐containing protein 1 OS = Homo sapiens OX = 9606 GN = ANKRD1	ANKRD1	0.74	Down	.0004	1.292	Up	.0006	Cyto_nucl

^a^ S/D: damage group versus control group, C/S: C3G intervention group for oxidative damage versus damage group.

### 3.4 C3G interfered with cholesterol synthesis in HEK‐293T cells under oxidative stress

**FIGURE 5:**
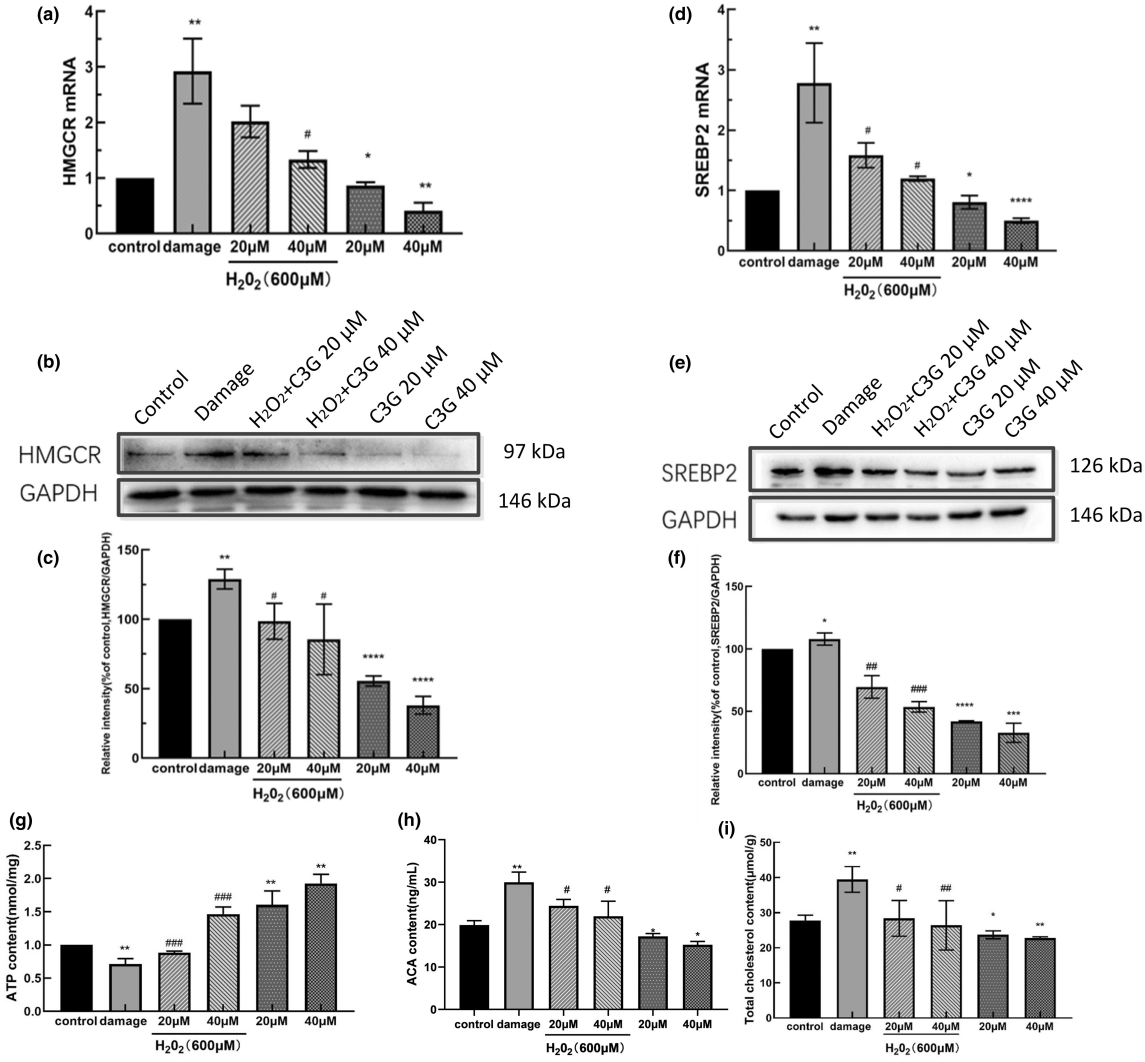
Effect of C3G on cholesterol synthesis in HEK‐293T cells under oxidative stress. Cells were pretreated with various concentrations of C3G (20 and 40 μM) for 12 h followed by treatment with or without H_2_O_2_ (600 μM) for 6 h. (a) The effects of C3G on HMGCR mRNA levels were analyzed by qRT‐PCR. (b, c) The effects of C3G on protein expressions of HMGCR were analyzed by Western Blot. (d) The effects of C3G on SREBP2 mRNA levels. (e, f) The effects of C3G on protein expressions of SREBP2. (g) The effects of C3G on ATP levels in oxidative stress cells. (h) The effects of C3G on the contents of acetyl‐CoA in oxidative stress cells. (i) The effects of C3G on total cholesterol levels in oxidative stress cells. The results were represented as means ± SD from experiments in triplicate. *p < .05, **p < .05, ***p < .001, versus control group; ^#^ *p <* .05, ^##^ *p <* .01, ^##^ *p <* .001 versus damage group.

The DEPs and upstream‐related regulators were verified by western blotting and qRT‐PCR. As shown in Figure 5, the mRNA and protein expressions of SREBP2 and HMGCR were upregulated after H_2_O_2_‐induced damage in HEK‐293T cells. However, the mRNA and protein expressions of SREBP2 and HMGCR in oxidation‐damaged cells pretreated with C3G were significantly downregulated compared to the damage group (*p* < .05) (Figure 5a–f). The above results were in line with the proteomic analysis. The proteins related to the regulation of cholesterol synthesis during the inhibition of cellular oxidative stress by C3G were successfully screened.

At the same time, the effects of C3G on the glycolysis pathway, raw material of cholesterol synthesis, and total cholesterol level were investigated. The decreased ATP level (Figure 5g), increased acetyl‐CoA level (Figure 5h), and intracellular cholesterol (Figure 5i) were detected in HEK‐293T cells when exposed to H_2_O_2_. However, it was found that C3G intervention in cell damage can significantly increase ATP levels and reduce the level of acetyl‐CoA and intracellular cholesterol (*p* < .05).

### 3.5 C3G modulates HMGCR‐induced ROS generation

Cell models of HMGCR knockdown and overexpression were established to further determine whether HMGCR is the target of cellular oxidative stress inhibited by C3G. The protein expression and mRNA level of HMGCR in HEK‐293T cells could be significantly knocked down by siRNA3 transfection (Figure S2A,C). In the overexpression test (Figure S2B,D), the expression levels of protein and mRNA in the overexpressed plasmid transfection group were significantly increased. Both siRNA knockdown and plasmid overexpression models were successfully established.

**FIGURE 6:**
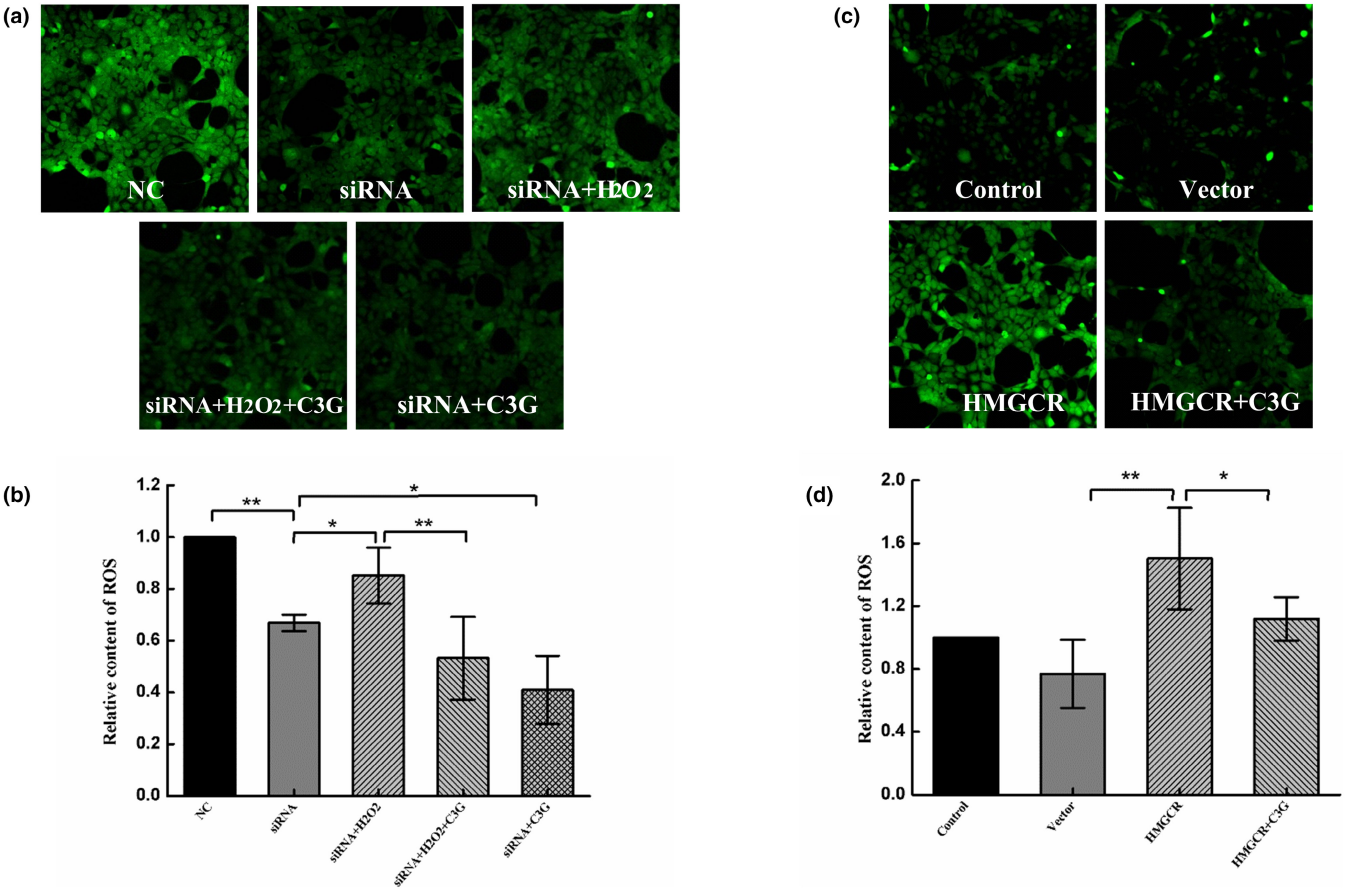
Effects of C3G on intracellular ROS production after siRNA knockdown and plasmid overexpression. HEK‐293T cells were treated with either HMGCR‐specific siRNA3 or plasmid for 24 h before treatment with C3G (40 μmol/L) for 12 h, and then exposed with or without H_2_O_2_ (600 μmol/L). (a) Fluorescence images of siRNA knockdown and C3G intervening oxidative damage were observed by a laser scanning confocal microscope. (b) ROS levels in the condition of HMGCR knockdown were analyzed by ImageJ. (c) Fluorescence images of plasmid overexpression and C3G intervention were observed by a laser scanning confocal microscope. (d) ROS levels in the condition of HMGCR overexpression were analyzed by ImageJ. The results were the means ± SD of three independent experiments. *p < .05 and **p < .01 between the two groups indicate significant differences.

**FIGURE 7:**
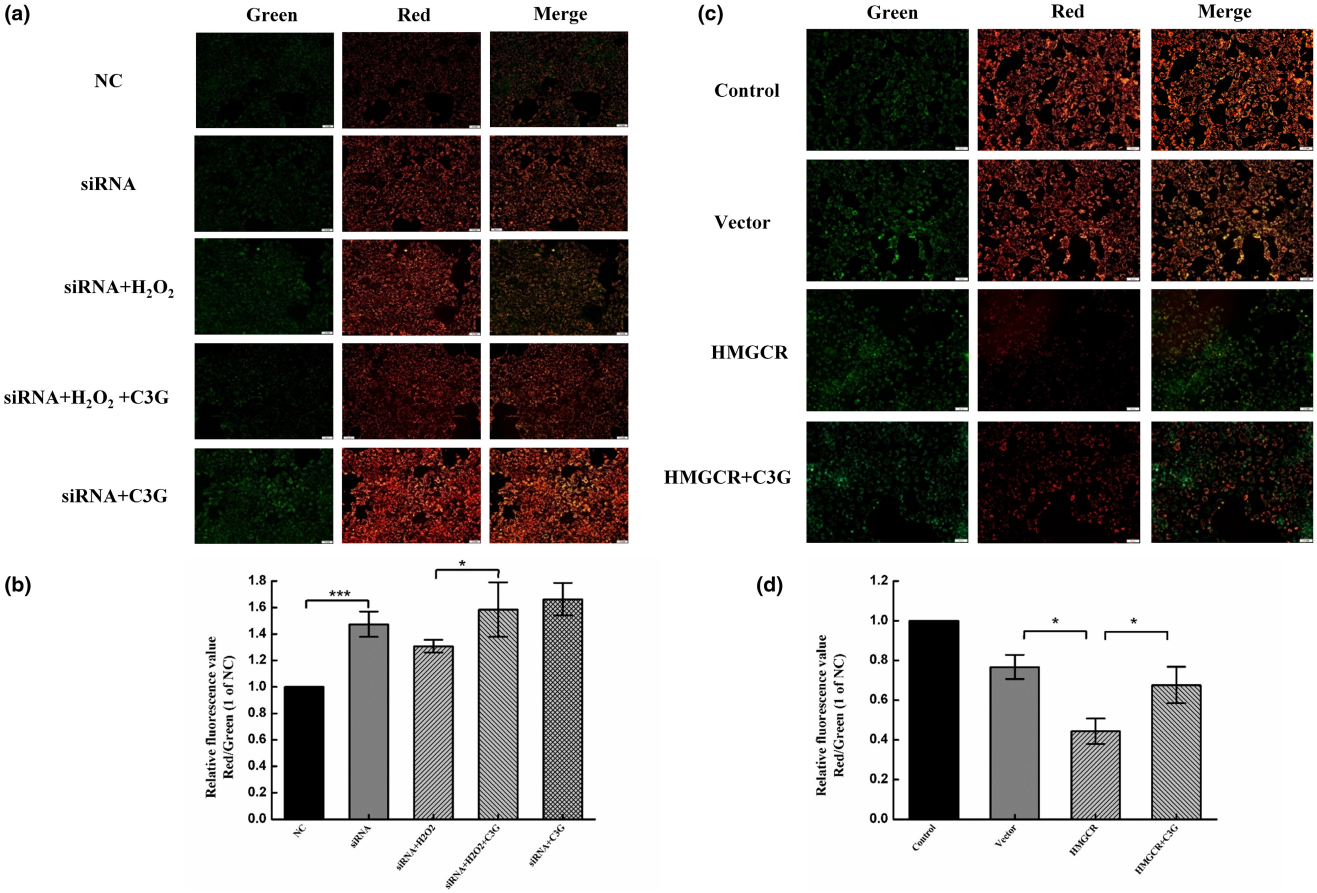
Effects of C3G on MMP after siRNA knockdown and plasmid overexpression. HEK‐293T cells were treated with either HMGCR‐specific siRNA3 or plasmid for 24 h before treatment with C3G (40 μmol/L) for 12 h, and then exposed with or without H_2_O_2_ (600 μmol/L). (a) Fluorescence images of siRNA knockdown and C3G intervening oxidative damage were observed by a fluorescence microscope. (b) MMP levels in the condition of HMGCR knockdown were analyzed by ImageJ. (c) Fluorescence images of plasmid overexpression and C3G intervention were observed by a fluorescence microscope. (d) MMP levels in the condition of HMGCR overexpression were analyzed by ImageJ. The results were the means ± SD of three independent experiments. *p < .05 and ***p < .001 between the two groups indicate significant differences.

Next, how HMGCR knockdown affects the production of ROS with or without the intervention of C3G was evaluated. As shown in Figure 7, the level of intracellular ROS was significantly decreased after knocking down by siRNA (Figure 6a,b). The level of ROS increased after H_2_O_2_ induction, while it was significantly decreased after C3G intervention. Indicating that part of the anti‐oxidative stress mechanism of C3G is the inhibition of ROS, but whether it is related to the inhibition of cholesterol synthesis needs further study. Therefore, HMGCR were overexpressed by plasmid transfection to investigate the relationship between ROS and cholesterol synthesis, and the intervention effect of C3G (Figure 6c,d). The results showed that overexpression of HMGCR could lead to an increase in ROS level, which was reduced by C3G treatment. The results confirmed that C3G could indeed inhibit ROS accumulation caused by excessive cholesterol synthesis, and thus resist oxidative damage.

### 3.6 C3G modulates HMGCR‐induced MMP changes

Meanwhile, we determined whether C3G‐protected mitochondrial membranes were related to regulating cholesterol synthesis. As shown in Figure 7, MMP was found to be increased when HMGCR was knocked down by siRNA transfection (Figure 7a,b), indicating that inhibition of cholesterol synthesis could protect mitochondria to a certain extent. MMP was decreased after H_2_O_2_ induction but significantly increased after C3G intervention in injured cells. These results indicated that C3G could protect the mitochondrial membrane against oxidative stress. So, whether this protective effect was related to the inhibition of cholesterol accumulation was investigated by overexpression assay. The results showed that overexpression of HMGCR could lead to a decrease in MMP, indicating that cholesterol accumulation could damage mitochondria. While C3G‐treated cells with overexpression could significantly increase MMP levels (Figure 7c,d), indicating that C3G could protect the mitochondrial membrane by inhibiting excessive cholesterol synthesis.

### 3.7 C3G interferes SREBP2/HMGCR pathway to regulate cholesterol synthesis

**FIGURE 8:**
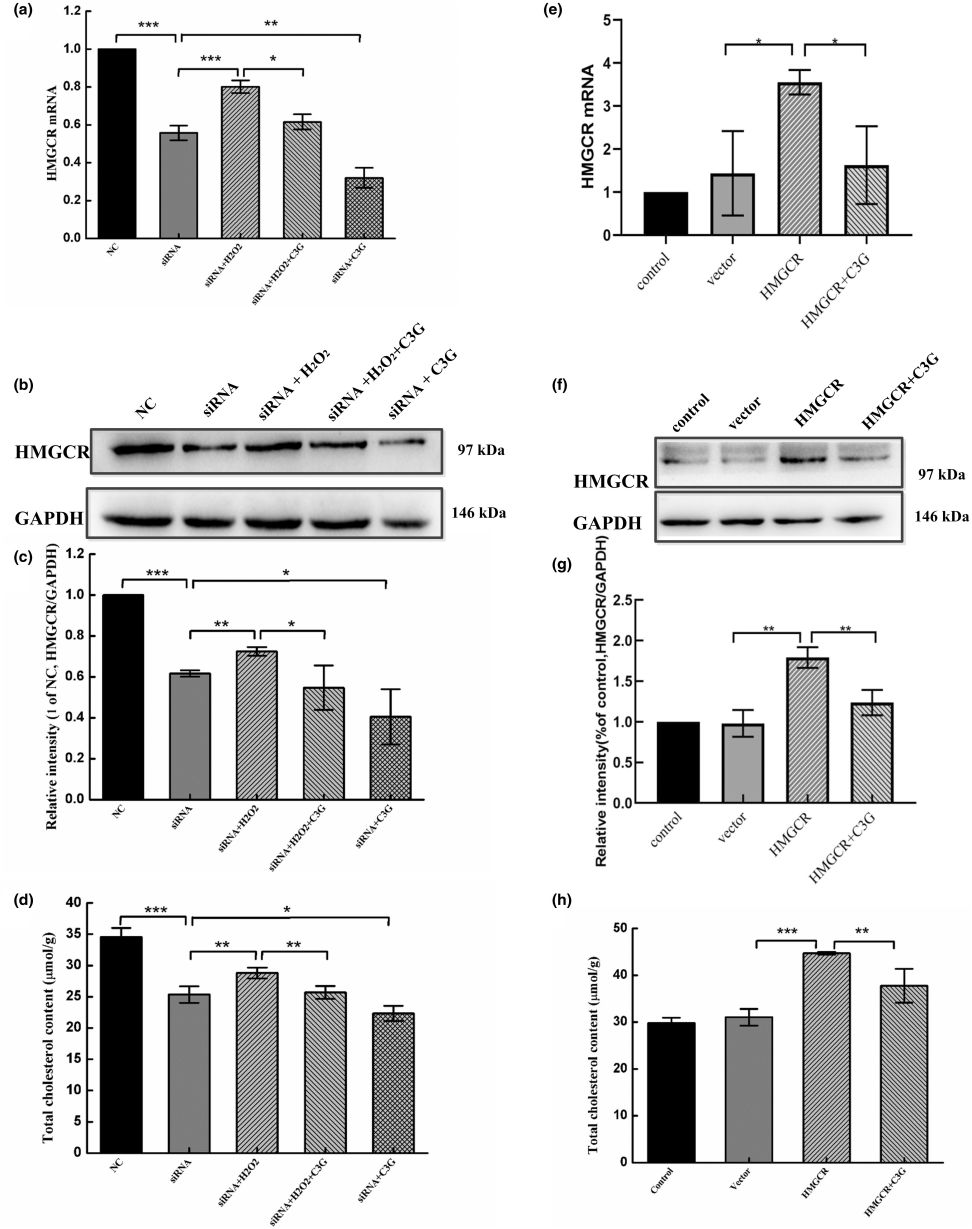
Effects of C3G on HMGCR expression and total cholesterol level after siRNA knockdown and plasmid overexpression. HEK‐293T cells were treated with either HMGCR‐specific siRNA3 or plasmid for 24 h before treatment with C3G (40 μmol/L) for 12 h, and then exposed with or without H_2_O_2_ (600 μmol/L). (a) The effects of C3G on HMGCR mRNA levels after knockdown of siRNA. (b, c) The effects of C3G on protein expressions of HMGCR after knockdown. (d) The effects of C3G on total cholesterol levels after knockdown of HMGCR. (e) The effects of C3G on HMGCR mRNA levels after overexpression of plasmid. (f, g) The effects of C3G on the protein expressions of HMGCR after overexpression. (h) The effects of C3G on total cholesterol levels after overexpression of HMGCR. The results are the means ± SD of three independent experiments. *p < .05, **p < .01 and ***p < .001 between two group indicate significant differences.

In order to explore the intervention mechanism of C3G on cholesterol synthesis in oxidative stress cells, the key protein of cholesterol synthesis, HMGCR, was knocked down and overexpressed to investigate the effects of C3G on HMGCR expression and total cholesterol level in cells. As shown in Figure 8, the mRNA level and protein expression of HMGCR were significantly upregulated after inducing by H_2_O_2_ in knockdown cells, as well as the cholesterol level. While C3G pretreatment could significantly reduce HMGCR expression and cholesterol levels in both ordinary knockdown cells and oxidative knockdown cells (Figure 8a–d). These results indicated that H_2_O_2_‐induced oxidative stress could lead to excessive cholesterol synthesis, while C3G intervention could significantly inhibit cholesterol synthesis and alleviate oxidative damage. In order to further confirm the positive effects of C3G, HMGCR was overexpressed by plasmid. It was found that cholesterol level was significantly increased. After C3G intervention, both HMGCR expression and cholesterol level were significantly decreased (Figure 8e–h).

## 4 DISCUSSION

Using proteomics and a series of in vitro gene intervention experiments, this study indicates for the first time that HMGCR‐mediated cholesterol synthesis plays a central role in the inhibition of oxidative stress progression by C3G. Firstly, the positive role of C3G in inhibiting cellular oxidative stress was identified. The differentially expressed protein, HMGCR was screened by proteomic analysis. And it was verified that C3G reduced cholesterol levels by interfering with SREBP2/HMGCR pathway, increasing ATP and reducing acetyl‐CoA. ROS production and MMP were evaluated through a series of knockdown and overexpression experiments to confirm the role of HMGCR in the anti‐cellular oxidative stress of C3G. Knockdown and overexpression tests were conducted to further confirm the mechanism of HMGCR in the regulation of cholesterol anabolism by C3G. Finally, it was found that C3G could inhibit the disorder of cholesterol synthesis in oxidative stress cells by regulating the ROS/SREBP2/HMGCR pathway, and play a role in ensuring accurate lipid renewal.

The reducing capacity and antioxidant capacity of C3G are significantly stronger than the natural antioxidants resveratrol, ascorbic acid, and other anthocyanin extracts (Sun et al., 2016). C3G exhibits a variety of biological activities, such as prevention and treatment of fatty liver, treatment of diabetes, anti‐tumor, anti‐inflammatory, anti‐thrombosis, and reproductive protection. However, these functions cannot be separated from the core role of antioxidant. Our previous study also demonstrated that C3G can regulate Nrf2/Keap1 signaling pathway to improve the activity of antioxidant enzymes GSH‐Px, SOD, and CAT (D. Liu et al., 2016). In this study, C3G increased cell survival following H_2_O_2_ injury (Figure 1c). C3G could partially suppress the cell injury by antioxidative function. While the cell viability could not revert to control level.

ROS directly damages the abundant lipids in cell biofilm and causes lipid peroxidation. Therefore, elimination of excess ROS through antioxidants may be a key way to inhibit cell biofilm damage. The present study demonstrated that H_2_O_2_ exposure markedly accelerates ROS production and MMP decrease in HEK‐293T cells, which were reversed by C3G intervention (Figure 2), suggesting that C3G has a protective effect on mitochondria against ROS‐induced oxidative stress. In addition, C3G can also effectively inhibit ROS in different cells under oxidative stress induced by H_2_O_2_ (Tan et al., 2020), which is consistent with the results of this experiment. Related studies have shown that C3G also inhibits lead‐induced ROS overproduction and decreases cellular progesterone biosynthesis (Sun et al., 2016; Wen et al., 2018). Pre‐treatment with C3G reduced the levels of intracellular ROS and lipid peroxidation in the MIN6N cells treated with H_2_O_2_ (Lee et al., 2015). Based on the above studies, we use proteomics to further screen for target proteins involved in the antioxidant effect of C3G.

According to GO enrichment analysis in proteomics, the functions of protein enrichment upregulated in the S/D group but downregulated in C/S group were related to cell membrane composition, cell stress and death, and cholesterol metabolism (Figure 3). PPI analysis of the DAPs between H_2_O_2_ treated and control groups demonstrated direct interactions among in sterol‐sensing and biosynthesis, DNA and protein synthesis, cell proliferation, and apoptosis, via HMGCR as connecting protein (Figure 4). However, PPI analysis of the C3G intervention group (vs. H_2_O_2_ treated group) found that another interaction such as anti‐oxidative stress appeared, indicating that the intervention of C3G was not only based on the interaction between sterols and biosynthesis linked by HMGCR but also has other antioxidant effects. The PPI network indicates that the mechanism of action of C3G is complex, the cross‐talk among DNA, protein, and lipid, and the interaction between HMGCR‐related signaling pathway and cell anabolic metabolism may be one of the main defense mechanisms to prevent H_2_O_2_‐induced oxidative damage of cells. Importantly, the analysis demonstrated that HMGCR protein level was dramatically elevated in H_2_O_2_‐induced oxidative cells and downregulated by C3G pretreatment. As a rate‐limiting enzyme in cholesterol biosynthesis, HMGCR is highly regulated at the transcriptional, translational, and post‐translational levels. SREBP2, the upstream regulator of HMGCR, is inserted into the ER as an inactive precursor and senses cholesterol levels by SREBP cleavage activating protein (SCAP) (Horton et al., 2002). Interestingly, proteomic analysis showed that SCAP was upregulated in the damage group and downregulated after C3G intervention (Table 1). Therefore, it can be speculated that the antioxidant effect of C3G may be related to the regulation of cholesterol anabolism.

In this study, C3G administration suppressed intervention in cell damage can increase the level of ATP, and reduce the level of SREBP2, HMGCR, and acetyl‐CoA (Figure 5). Acetyl‐CoA is a precursor of cholesterol synthesis. Mitochondrial acetyl‐CoA can enter the tricarboxylic acid (TCA) cycle, thereby generating ATP and reducing equivalents (Houston et al., 2020). It indicates that C3G can reduce the generation of cholesterol raw material by inhibiting Acetyl‐CoA accumulation. Studies have revealed hypoxia‐induced metabolic switches that divert glucose metabolites from mitochondria to glycolysis to maintain ATP production and prevent the production of toxic ROS (Kim et al., 2006). The present study demonstrated that H_2_O_2_ exposure markedly suppresses ATP and accelerates Acetyl‐CoA production in HEK‐293T cells. Combined with the above results, it can be speculated that C3G can promote acetyl‐CoA to participate in the tricarboxylic acid cycle to produce ATP by repairing mitochondrial function. Thus, the glycolysis pathway was inhibited, the accumulation of raw materials for cholesterol synthesis was reduced, and the de novo synthesis of cholesterol was inhibited. Relevant studies have also confirmed that excessive ROS will increase glucose uptake, promote the glycolysis process, and reduce the production of ATP (Seo et al., 2019). Anthocyanin suppressed Acetyl‐CoA activity in a dose‐dependent fashion in HepG2 cells (Guo et al., 2012). This is consistent with the experimental results. The results also showed that the intracellular cholesterol level in oxidation‐damaged cells could be reduced by C3G intervention (Figure 3). These results demonstrated that C3G significantly inhibited the production and accumulation of cholesterol, and maintained the balance of total cholesterol level in the process of reducing the oxidative stress.

Excessive ROS will increase the uptake and accumulation of cholesterol, destroy mitochondrial function, and cause cell damage. A series of knockdown and overexpression experiments were used to investigate whether the regulation of C3G on ROS level is related to HMGCR‐mediated cholesterol synthesis. Correspondingly, the ROS level was prevented by siRNA of HMGCR, and promoted by overexpressing plasmid of HMGCR, suggesting a positive correlation between HMGCR activity and ROS elevation (Figure 6a,c). In this study, excessive ROS generation induced by H_2_O_2_ in HMGCR knockdown cells was lowered by the C3G treatment (Figure 6c,d). Moreover, the elevated ROS induced by HMGCR overexpress was also reduced by C3G treatment (Figure 6d). ROS generation during purine metabolism increased cholesterol accumulation and decreased APOE and ABCA1 expression (Ryu et al., 2016). Inversely, cholesterol and oxysterols could cause ROS production (Anavi et al., 2014; Poli et al., 2013). Altogether, these observations support the notion that modulation of ROS by C3G intervention is closely associated with HMGCR‐mediated cholesterol synthesis.

Excessive cholesterol can lead to mitochondrial dysfunction which is characterized by the loss of mitochondrial membrane integrity, ROS overproduction, and apoptosis (Farnaghi et al., 2017). Thus, the balance between ROS overproduction and cholesterol homeostasis is the key to maintaining normal mitochondrial function. In this study, the relationship between the protective effect of C3G on mitochondria and the regulation of cholesterol metabolism was further revealed. As expected, HMGCR knockdown led to an elevation of MMP, while the overexpression of HMGCR decreased MMP to some extent (Figure 7b,d). The MMP of HMGCR knockdown cells was decreased after exposure to H_2_O_2_ with no significance, while C3G enhanced the MMP activity of HEK‐293T cells at a certain concentration. HMGCR overexpression experiment showed that C3G could directly promote the increase of MMP (Figure 7). The results demonstrated that C3G enhances MMP and inhibits mitochondrial dysfunction, primarily by reducing HMGCR‐induced cholesterol increases. These data suggest that, on the one hand, overexpression of HMGCR is at least partly responsible for an increase in ROS and a decrease in MMP, similar to the results of H_2_O_2_ exposure. On the other hand, HMGCR‐mediated cholesterol synthesis is involved in the changes of ROS and MMP during the regulation of cellular oxidative stress by C3G. We conclude that HMGCR is an essential regulator involved in the resistance of C3G to oxidative stress.

The cholesterol and phospholipids in cell membranes are highly susceptible to ROS‐induced peroxidation (Jurcau et al., 2022), which leads to lipid peroxidation, cell membrane damage, and cell death through a series of free radical chain reactions (Mao et al., 2022; Nury et al., 2021). Thus, the maintenance of cellular cholesterol homeostasis is the key to regulate the accurate lipid renewal of cells, protect cell biofilm, and resist oxidative damage. The present study indicated that H_2_O_2_ could still activate the expression of HMGCR and lead to excessive cholesterol synthesis under the condition of HMGCR knockdown. The intervention of C3G could significantly reduce the expression of HMGCR and inhibit cholesterol synthesis with both exposed and unexposed to H_2_O_2_ in knockdown cells (Figure 8). In addition, C3G could also reduce expression levels and cholesterol levels in the case of HMGCR overexpression. This confirmed the role of HMGCR in mediated cholesterol synthesis in the inhibition of H_2_O_2_‐induced cellular oxidative stress by C3G. The antioxidant research related to cell metabolism disorders has gradually received attention in these years. The anthocyanin extract of strawberries could lead to phosphorylation and inactivation of acetyl‐CoA carboxylase by activating AMPK, thereby inhibiting HMGCR and cholesterol synthesis (Forbes‐Hernandez et al., 2017). In addition, anthocyanins could inhibit oxidative stress and reduce inflammatory cytokines by activating the tyrosine metabolic pathway to a certain extent (Li et al., 2022). C3G and PCA appeared to promote cholesterol outflow and HDL formation by activating the liver X receptor (LXR) and/or regulating lipid transporters, including ABCA1 and ABCG1 (Jia, Hoang, et al., 2013; Jia, Kim, et al., 2013; Wang et al., 2012). Based on this, the present study demonstrated that the mechanism by which C3G interferes with the excessive synthesis of cholesterol and inhibits oxidative stress regulates the transcription and expression of HMGCR.

In vivo study revealed that the high‐fat‐diet (HFD) increased MDA and CAT levels and decreased GSH and SOD levels in mice, which meant the oxidative stress was significantly increased by HFD. While this phenomenon was reversed by C3G, which alleviated the oxidative stress level and played a good antioxidant role in HFD mice (Lyu et al., 2023). C3G could reduce total cholesterol (TC), triglycerides (TG), and low‐density lipoprotein cholesterol (LDL‐C) and alanine and aspartate aminotransferase (ALT and AST) levels in serum, and enhance high‐density lipoprotein (HDL‐C) levels in hyperlipidemia rats and bisphenol A‐induced liver lipid metabolism disorder rats (Liu et al., 2023; Wu et al., 2016). C3G effectively facilitated the recovery of differential lipid metabolites and reversed the levels of hepatic lipid synthesis‐related genes (Liu et al., 2023). The above studies indicate that C3G can also maintain cholesterol homeostasis in vivo, play a precise role in lipid regulation, and is associated with improving oxidative stress. It can be inferred that C3G may play a role in regulating cholesterol anabolism in vivo by targeting HMGCR.

In summary, well‐balanced redox status and stable cellular cholesterol metabolism are the key factors to regulate the accurate renewal of lipids and maintain cell physiological functions. Our quantitative proteomics study has provided a new perspective and a new direction for the antioxidant research of C3G. C3G can reduce cholesterol synthesis by reducing the expression of SREBP2 and HMGCR, thereby maintaining cholesterol levels and exerting an antioxidant effect by regulating cholesterol homeostasis. This study not only revealed that HMGCR‐mediated cholesterol synthesis is related to cellular redox status but also confirmed the role of cholesterol synthesis regulated by C3G in reducing ROS accumulation and protecting mitochondria, and finally identified HMGCR as a target for inhibiting oxidative stress mediated by C3G. However, this study also has some limitations, the regulatory effects of C3G on cholesterol synthesis and transport should be further verified in vivo.

## AUTHOR CONTRIBUTIONS

**Di Liu:** Conceptualization (lead); data curation (equal); funding acquisition (equal); investigation (equal); writing – original draft (lead). **Hanxue Zhang:** Data curation (equal); investigation (equal). **Yu Dai:** Validation (equal). **Jie Sun:** Methodology (lead); visualization (equal). **Hongyu Sun:** Formal analysis (lead); resources (equal). **Zixiang Yu:** Software (lead); validation (equal). **Fanli Kong:** Supervision (equal); writing – review and editing (equal). **Xianmin Feng:** Project administration (lead); supervision (equal); writing – review and editing (equal).

## FUNDING INFORMATION

The authors acknowledge the financial supports of the Natural Science Foundation of Jilin Province (No. 20200201146JC, 20220101307JC) and the Science and Technology Research Project of Jilin Provincial Department of Education (No. JJKH20210053KJ).

## CONFLICT OF INTEREST STATEMENT

The authors have declared no conflict of interest.

## Supporting information


Data S1

## Data Availability

The data that support the findings of this study are available from the corresponding author upon reasonable request.

## References

[fsn34231-bib-0001] Ajit, D. , Simonyi, A. , Li, R. , Chen, Z. , Hannink, M. , Fritsche, L. K. , Smith, R. E. , Dobbs, T. K. , Luo, R. , Folk, W. R. , Gu, Z. , Lubahn, D. B. , Weisman, G. A. , & Sun, Y. G. (2016). Phytochemicals and botanical extracts regulate NF‐κB and Nrf2/ARE reporter activities in DI TNC1 astrocytes. Neurochemistry International, 97, 49–56. 10.1016/j.neuint.2016.05.004 27166148 PMC4900906

[fsn34231-bib-0002] Anavi, S. , Hahn‐Obercyger, M. , Madar, Z. , & Tirosh, O. (2014). Mechanism for HIF‐1 activation by cholesterol under normoxia: A redox signaling pathway for liver damage. Free Radical Biology & Medicine, 71, 61–69. 10.1016/j.freeradbiomed.2014.03.007 24632196

[fsn34231-bib-0003] Farnaghi, S. , Prasadam, I. , Cai, G. , Friis, T. , Du, Z. , Crawford, R. , Mao, X. , & Xiao, Y. (2017). Protective effects of mitochondria‐targeted antioxidants and statins on cholesterol‐induced osteoarthritis. The FASEB Journal, 31(1), 356–367. 10.1096/fj.201600600R 27737897

[fsn34231-bib-0004] Ferrari, D. , Cimino, F. , Fratantonio, D. , Molonia, S. M. , Bashllari, R. , Busà, R. , Saija, A. , & Speciale, A. (2017). Cyanidin‐3‐O‐glucoside modulates the in vitro inflammatory crosstalk between intestinal epithelial and endothelial cells. Mediators of Inflammation, 2017, 3454023. 10.1155/2017/3454023 28373746 PMC5360945

[fsn34231-bib-0005] Ferreira, F. S. , de Oliveira, V. S. , Chavez, D. W. H. , Chaves, D. S. , Riger, C. J. , Sawaya, A. , Guizellini, G. M. , Sampaio, G. R. , Torres, E. , & Saldanha, T. (2022). Bioactive compounds of parsley (*Petroselinum crispum*), chives (*Allium schoenoprasum* L) and their mixture (Brazilian cheiro‐verde) as promising antioxidant and anti‐cholesterol oxidation agents in a food system. Food Research International, 151, 110864. 10.1016/j.foodres.2021.110864 34980400

[fsn34231-bib-0006] Forbes‐Hernandez, T. Y. , Giampieri, F. , Gasparrini, M. , Afrin, S. , Mazzoni, L. , Cordero, M. D. , Mezzetti, B. , Quiles, J. L. , & Battino, M. (2017). Lipid accumulation in HepG2 cells is attenuated by strawberry extract through AMPK activation. Nutrients, 9(6), 621. 10.3390/nu9060621 28621732 PMC5490600

[fsn34231-bib-0007] Frey, L. , Hiller, S. , Riek, R. , & Bibow, S. (2018). Lipid‐ and cholesterol‐mediated time‐scale‐specific modulation of the outer membrane protein X dynamics in lipid bilayers. Journal of the American Chemical Society, 140(45), 15402–15411. 10.1021/jacs.8b09188 30289706

[fsn34231-bib-0008] Gaschler, M. M. , & Stockwell, B. R. (2017). Lipid peroxidation in cell death. Biochemical and Biophysical Research Communications, 482(3), 419–425. 10.1016/j.bbrc.2016.10.086 28212725 PMC5319403

[fsn34231-bib-0009] Guo, H. , Liu, G. , Zhong, R. , Wang, Y. , Wang, D. , & Xia, M. (2012). Cyanidin‐3‐O‐β‐glucoside regulates fatty acid metabolism via an AMP‐activated protein kinase‐dependent signaling pathway in human HepG2 cells. Lipids in Health and Disease, 11, 10. 10.1186/1476-511x-11-10 22243683 PMC3398342

[fsn34231-bib-0010] Horton, D. J. , Goldstein, L. J. , & Brown, S. M. (2002). SREBPs: activators of the complete program of cholesterol and fatty acid synthesis in the liver. The Journal of Clinical Investigation, 109(9), 1125–1131. 10.1172/JCI15593 11994399 PMC150968

[fsn34231-bib-0011] Houston, R. , Sekine, S. , Calderon, M. J. , Seifuddin, F. , Wang, G. , Kawagishi, H. , Malide, D. A. , Li, Y. , Gucek, M. , Pirooznia, M. , Nelson, A. J. , Stokes, M. P. , Stewart‐Ornstein, J. , Mullett, S. J. , Wendell, S. G. , Watkins, S. C. , Finkel, T. , & Sekine, Y. (2020). Acetylation‐mediated remodeling of the nucleolus regulates cellular acetyl‐CoA responses. PLoS Biology, 18(11), e3000981. 10.1371/journal.pbio.3000981 33253182 PMC7728262

[fsn34231-bib-0012] Jia, Y. , Hoang, M. H. , Jun, H. J. , Lee, J. H. , & Lee, S. J. (2013). Cyanidin, a natural flavonoid, is an agonistic ligand for liver X receptor alpha and beta and reduces cellular lipid accumulation in macrophages and hepatocytes. Bioorganic & Medicinal Chemistry Letters, 23(14), 4185–4190. 10.1016/j.bmcl.2013.05.030 23769638

[fsn34231-bib-0013] Jia, Y. , Kim, J. Y. , Jun, H. J. , Kim, S. J. , Lee, J. H. , Hoang, M. H. , Kim, H. S. , Chang, H. I. , Hwang, K. Y. , Um, S. J. , & Lee, S. J. (2013). Cyanidin is an agonistic ligand for peroxisome proliferator‐activated receptor‐alpha reducing hepatic lipid. Biochimica et Biophysica Acta, 1831(4), 698–708. 10.1016/j.bbalip.2012.11.012 23228689

[fsn34231-bib-0014] Jurcau, M. C. , Andronie‐Cioara, F. L. , Jurcau, A. , Marcu, F. , Tit, D. M. , Pascalau, N. , & Nistor‐Cseppento, D. C. (2022). The link between oxidative stress, mitochondrial dysfunction and neuroinflammation in the pathophysiology of Alzheimer's disease: Therapeutic implications and future perspectives. Antioxidants (Basel), 11(11), 2167. 10.3390/antiox11112167 36358538 PMC9686795

[fsn34231-bib-0015] Kamiloglu, S. , Capanoglu, E. , Grootaert, C. , & Camp, V. J. (2015). Anthocyanin absorption and metabolism by human intestinal Caco‐2 cells‐a review. International Journal of Molecular Sciences, 16(9), 21555–21574. 10.3390/ijms160921555 26370977 PMC4613267

[fsn34231-bib-0016] Kim, J. , Tchernyshyov, I. , Semenza, G. , & Dang, C. (2006). HIF‐1‐mediated expression of pyruvate dehydrogenase kinase: a metabolic switch required for cellular adaptation to hypoxia. Cell Metabolism, 3(3), 177–185. 10.1016/j.cmet.2006.02.002 16517405

[fsn34231-bib-0017] Lee, J. S. , Kim, Y. R. , Song, I. G. , Ha, S. J. , Kim, Y. E. , Baek, N. I. , & Hong, E. K. (2015). Cyanidin‐3‐glucoside isolated from mulberry fruit protects pancreatic β‐cells against oxidative stress‐induced apoptosis. International Journal of Molecular Medicine, 35(2), 405–412. 10.3892/ijmm.2014.2013 25435295

[fsn34231-bib-0018] Li, F. , Wang, Y. , Li, Y. , Yang, H. , & Wang, H. (2018). Quantitative analysis of the global proteome in peripheral blood mononuclear cells from patients with new‐onset psoriasis. Proteomics, 18(19), e1800003. 10.1002/pmic.201800003 30094923

[fsn34231-bib-0019] Li, T. , Yang, Y. , Wang, X. , Dai, W. , Zhang, L. , & Piao, C. (2021). Flavonoids derived from buckwheat hull can break advanced glycation end‐products and improve diabetic nephropathy. Food & Function, 12(16), 7161–7170. 10.1039/d1fo01170g 34169956

[fsn34231-bib-0020] Li, X. , Zhao, J. , Yan, T. , Mu, J. , Lin, Y. , Chen, J. , Deng, H. , & Meng, X. (2021). Cyanidin‐3‐O‐glucoside and cisplatin inhibit proliferation and downregulate the PI3K/AKT/mTOR pathway in cervical cancer cells. Journal of Food Science, 86(6), 2700–2712. 10.1111/1750-3841.15740 33908630

[fsn34231-bib-0021] Li, Y. , Lu, Y. , Tang, D. , Hu, B. , Zhang, Z. , Wu, H. , Fan, L. , Cai, K. , Tang, C. , Zhang, Y. , Hong, L. , Dong, J. , Guan, B. , Yin, L. , Dai, Y. , Bai, W. , Zheng, Z. , & Zhu, T. (2022). Anthocyanin improves kidney function in diabetic kidney disease by regulating amino acid metabolism. Journal of Translational Medicine, 20(1), 510. 10.1186/s12967-022-03717-9 36335368 PMC9636632

[fsn34231-bib-0022] Lila, M. A. , Burton‐Freeman, B. , Grace, M. , & Kalt, W. (2016). Unraveling anthocyanin bioavailability for human health. Annual Review of Food Science and Technology, 7, 375–393. 10.1146/annurev-food-041715-033346 26772410

[fsn34231-bib-0023] Liu, D. , Pan, F. , Liu, J. , Wang, Y. , Zhang, T. , Wang, E. , & Liu, J. (2016). Individual and combined antioxidant effects of ginsenoside F2 and cyanidin‐3‐O‐glucoside in human embryonic kidney 293 cells. RSC Advances, 6(84), 81092–81100. 10.1039/c6ra14831j

[fsn34231-bib-0024] Liu, R. , Jin, Y. , Liu, B. , Zhang, Q. , Li, X. , Cai, D. , Tian, L. , Jiang, X. , Zhang, W. , Sun, J. , & Bai, W. (2023). Untargeted lipidomics revealed the protective effects of cyanidin‐3‐O‐glucoside on bisphenol a‐induced liver lipid metabolism disorder in rats. Journal of Agricultural and Food Chemistry, 71(2), 1077–1090. 10.1021/acs.jafc.2c06849 36597173

[fsn34231-bib-0025] Lyu, Q. , Deng, H. , Wang, S. , El‐Seedi, H. , Cao, H. , Chen, L. , & Teng, H. (2023). Dietary supplementation with casein/cyanidin‐3‐O‐glucoside nanoparticles alters the gut microbiota in high‐fat fed C57BL/6 mice. Food Chemistry, 412, 135494. 10.1016/j.foodchem.2023.135494 36736183

[fsn34231-bib-0026] Mao, C. , Lei, G. , Horbath, A. , & Gan, B. (2022). Assessment of lipid peroxidation in irradiated cells. Methods in Cell Biology, 172, 37–50. 10.1016/bs.mcb.2022.05.003 36064225 PMC11881802

[fsn34231-bib-0027] Matés, J. M. , Segura, J. A. , Alonso, F. J. , & Márquez, J. (2008). Intracellular redox status and oxidative stress: implications for cell proliferation, apoptosis, and carcinogenesis. Archives of Toxicology, 82(5), 273–299. 10.1007/s00204-008-0304-z 18443763

[fsn34231-bib-0028] Nury, T. , Zarrouk, A. , Yammine, A. , Mackrill, J. J. , Vejux, A. , & Lizard, G. (2021). Oxiapoptophagy: A type of cell death induced by some oxysterols. British Journal of Pharmacology, 178(16), 3115–3123. 10.1111/bph.15173 32579703

[fsn34231-bib-0029] Poli, G. , Biasi, F. , & Leonarduzzi, G. (2013). Oxysterols in the pathogenesis of major chronic diseases. Redox Biology, 1(1), 125–130. 10.1016/j.redox.2012.12.001 24024145 PMC3757713

[fsn34231-bib-0030] Ryu, H. M. , Kim, Y. J. , Oh, E. J. , Oh, S. H. , Choi, J. Y. , Cho, J. H. , Kim, C. D. , Park, S. H. , & Kim, Y. L. (2016). Hypoxanthine induces cholesterol accumulation and incites atherosclerosis in apolipoprotein E‐deficient mice and cells. Journal of Cellular and Molecular Medicine, 20(11), 2160–2172. 10.1111/jcmm.12916 27396856 PMC5082407

[fsn34231-bib-0031] Seo, E. , Kang, H. , Choi, H. , Choi, W. , & Jun, H. S. (2019). Reactive oxygen species‐induced changes in glucose and lipid metabolism contribute to the accumulation of cholesterol in the liver during aging. Aging Cell, 18(2), e12895. 10.1111/acel.12895 30609251 PMC6413652

[fsn34231-bib-0032] Sun, J. , Xu, W. , Zhu, C. , Hu, Y. , Jiang, X. , Ou, S. , Su, Z. , Huang, Y. , Jiao, R. , & Bai, W. (2016). Cyanidin‐3‐O‐Glucoside protects against 1,3‐dichloro‐2‐propanol‐induced reduction of progesterone by up‐regulation of steroidogenic enzymes and cAMP level in leydigcells. Frontiers in Pharmacology, 7, 399. 10.3389/fphar.2016.00399 27867356 PMC5096419

[fsn34231-bib-0033] Tan, J. , Li, P. , Xue, H. , & Li, Q. (2020). Cyanidin‐3‐glucoside prevents hydrogen peroxide (H(2)O(2))‐induced oxidative damage in HepG2 cells. Biotechnology Letters, 42(11), 2453–2466. 10.1007/s10529-020-02982-2 32780285

[fsn34231-bib-0034] van der Pol, A. , van Gilst, W. H. , Voors, A. A. , & van der Meer, P. (2019). Treating oxidative stress in heart failure: Past, present and future. European Journal of Heart Failure, 21(4), 425–435. 10.1002/ejhf.1320 30338885 PMC6607515

[fsn34231-bib-0035] Wang, D. , Xia, M. , Yan, X. , Li, D. , Wang, L. , Xu, Y. , Jin, T. , & Ling, W. (2012). Gut microbiota metabolism of anthocyanin promotes reverse cholesterol transport in mice via repressing miRNA‐10b. Circulation Research, 111(8), 967–981. 10.1161/circresaha.112.266502 22821931

[fsn34231-bib-0036] Wen, L. , Jiang, X. , Sun, J. , Li, X. , Li, X. , Tian, L. , Li, Y. , & Bai, W. (2018). Cyanidin‐3‐O‐glucoside promotes the biosynthesis of progesterone through the protection of mitochondrial function in Pb‐exposed rat leydig cells. Food and Chemical Toxicology, 112, 427–434. 10.1016/j.fct.2017.10.008 29030260

[fsn34231-bib-0037] Wu, T. , Yin, J. , Zhang, G. , Long, H. , & Zheng, X. (2016). Mulberry and cherry anthocyanin consumption prevents oxidative stress and inflammation in diet – induced obese mice. Molecular Nutrition & Food Research, 60(3), 687–694. 10.1002/mnfr.201500734 26627062

[fsn34231-bib-0038] Zarneshan, S. N. , Fakhri, S. , & Khan, H. (2022). Targeting Akt/CREB/BDNF signaling pathway by ginsenosides in neurodegenerative diseases: A mechanistic approach. Pharmacological Research, 177, 106099. 10.1016/j.phrs.2022.106099 35092819

[fsn34231-bib-0039] Zhao, L. , Pan, F. , Zhou, N. , Zhang, H. , Wang, Y. , Hao, S. , & Wang, C. (2021). Quantitative proteomics and bioinformatics analyses reveal the protective effects of cyanidin‐3‐O‐glucoside and its metabolite protocatechuic acid against 2‐amino‐3‐methylimidazo[4,5‐f]quinoline (IQ)‐induced cytotoxicity in HepG2 cells via apoptosis‐related pathways. Food and Chemical Toxicology, 153, 112256. 10.1016/j.fct.2021.112256 33974948

[fsn34231-bib-0040] Zhou, S. , Yin, X. , Yuan, J. , Liang, Z. , Song, J. , Li, Y. , Peng, C. , Hylands, P. J. , Zhao, Z. , & Xu, Q. (2022). Antifibrotic activities of scutellariae radix extracts and flavonoids: Comparative proteomics reveals distinct and shared mechanisms. Phytomedicine, 100, 154049. 10.1016/j.phymed.2022.154049 35397287

