# Supplementary material for: Cyanidin‐3‐O‐glucoside ameliorates hydrogen peroxide‐induced oxidative stress by regulating HMGCR‐mediated cholesterol anabolism in HEK‐293T cells
Source: Food Sci Nutr. 2024 Jul 1;12(9):6673–89. doi: 10.1002/fsn3.4231 (PMC11561815; doi:10.1002/fsn3.4231)
Supplement: Supplementary file 1 — Data S1 [file FSN3-12-6673-s001.docx]

**Supplementary Meterials**

Cyanidin-3-O-glucoside ameliorates hydrogen peroxide induced oxidative stress by regulating HMGCR mediated cholesterol anabolism in HEK-293T cells

Di Liu^1#^, Hanxue Zhang^2, 3#^, Yu Dai^1^, Jie Sun^1^, Hongyu Sun^1^, Zixiang Yu^1^, Fanli Kong^2,^ *, Xianmin Feng ^1,^ *

^1^ College of Basic Medicine, Jilin Medical University, Jilin, China

^2^ College of Medical Technology, Beihua University, Jilin, China

^3^ Department of Clinical Laboratory, Suzhou Hospital of Traditional Chinese Medicine, Suzhou, China

^#^These authors contributed equally to this work.

^*^ Corresponding Author: Xianmin Feng, E-mail: fengxianmin28@163.com

Fanli Kong, E-mail: kongfanli5@163.com

**Contents**

**Supplementary methods**

1. HPLC Fractionation

2. LC-MS/MS Analysis

3. Database Search

**Supplementary Figures and Tables**

Supplementary Figure 1. Proteomic analysis of HEK-293T oxygen-damaged cells pretreated with C3G.

Supplementary Figure 2. Transfection effects of different siRNA sequences and overexpressed plasmid in HEK-293T cells.

Supplementary Table 1. DEPs that expressed in damage group *vs* control group.

Supplementary Table 2. DEPs that expressed in C3G intervention group *vs* damage group.

**Supplementary methods**

**1. HPLC Fractionation**

The tryptic peptides were fractionated into fractions by high pH reverse-phase HPLC using Agilent 300Extend C18 column (5 μm particles, 4.6 mm diameter, 250 mm length). Briefly, the peptides were graded with 8%-32% acetonitrile (pH 9.0) within 60 minutes to obtain 60 components. Then, the peptides were combined into 18 fractions and freeze-dried under vacuum.

**2. LC-MS/MS Analysis**

The peptides were dissolved in 0.1% FA (solvent A) and separated by EASY-nLC 1000 UPLC. The elution gradient was set to 9%-25% solvent B (0.1% FA in 98% ACN) for 26 min, 25%-36% from 26 to 34 min, 36%-80% from 34 to 37 min, 80% from 37 to 40 min, and the flow rate maintained at 700 nL/min.

The gradient was comprised of an increase from 6% to 23% B (0.1% FA in 98% ACN) over 26 min, 23% to 35% in 8 min and climbing to 80% in 3 min then holding at 80% for the last 3 min, all at a constant flow rate of 400 nL/min on an EASY-nLC 1000 UPLC system.

The peptides were subjected to NSI source followed by tandem mass spectrometry (MS/MS) in Q ExactiveTM Plus (Thermo, Waltham, MA, USA) coupled online to the UPLC. The electrospray voltage applied was 2.0 kV. The m/z scan range of primary mass spectrometry was 350 to 1550 for full scan, and intact peptides were detected in the Orbitrap at a resolution of 60,000. Peptides were then selected for MS/MS using NCE setting as 28 and the fragments were detected in the Orbitrap at a resolution of 17,500. A data-dependent procedure that alternated between one MS scan followed by 20 MS/MS scans with 15.0s dynamic exclusion. Automatic gain control (AGC) was set at 5E4. Fixed first mass was set as 100 m/z.

**3. Database Search**

The resulting MS/MS data were processed using Maxquant search engine (v.1.5.2.8). Tandem mass spectra were searched against *SwissProt Human (20317 sequence)* database concatenated with reverse decoy database. Trypsin/P was specified as cleavage enzyme allowing up to 2 missing cleavages. The mass tolerance for precursor ions was set as 20 ppm in First search and 5 ppm in Main search, and the mass tolerance for fragment ions was set as 0.02 Da. Carbamidomethyl on Cys was specified as fixed modification and oxidation on Met was specified as variable modifications. The quantitative method was set to MTT-6plex, and the FDR for protein identification and PSM identification was set to 1%.

**Supplementary Figures**

**Supplementary Figure 1.** Proteomic analysis of HEK-293T oxygen-damaged cells pretreated with C3G. Cells were pretreated with various concentrations of C3G (20 μM and 40 μM) for 12 h fowllowed by treatment of H_2_O_2_ (600 μM) for 6 h. (A-F) GO enrichment bubble plot of DEPs in three categories. Downregulated DEPs of S/D enriched in Biological Process (A), Cellular Component (B), Molecular Function (C); Upregulated DEPs of C/S enriched in Biological Process (D), Cellular Component (E), Molecular Function (F). The results were analyzed from experiments in triplicate. S/D: damage group *vs* control group, C/S: C3G intervention group for oxidative damage *vs* damage group.


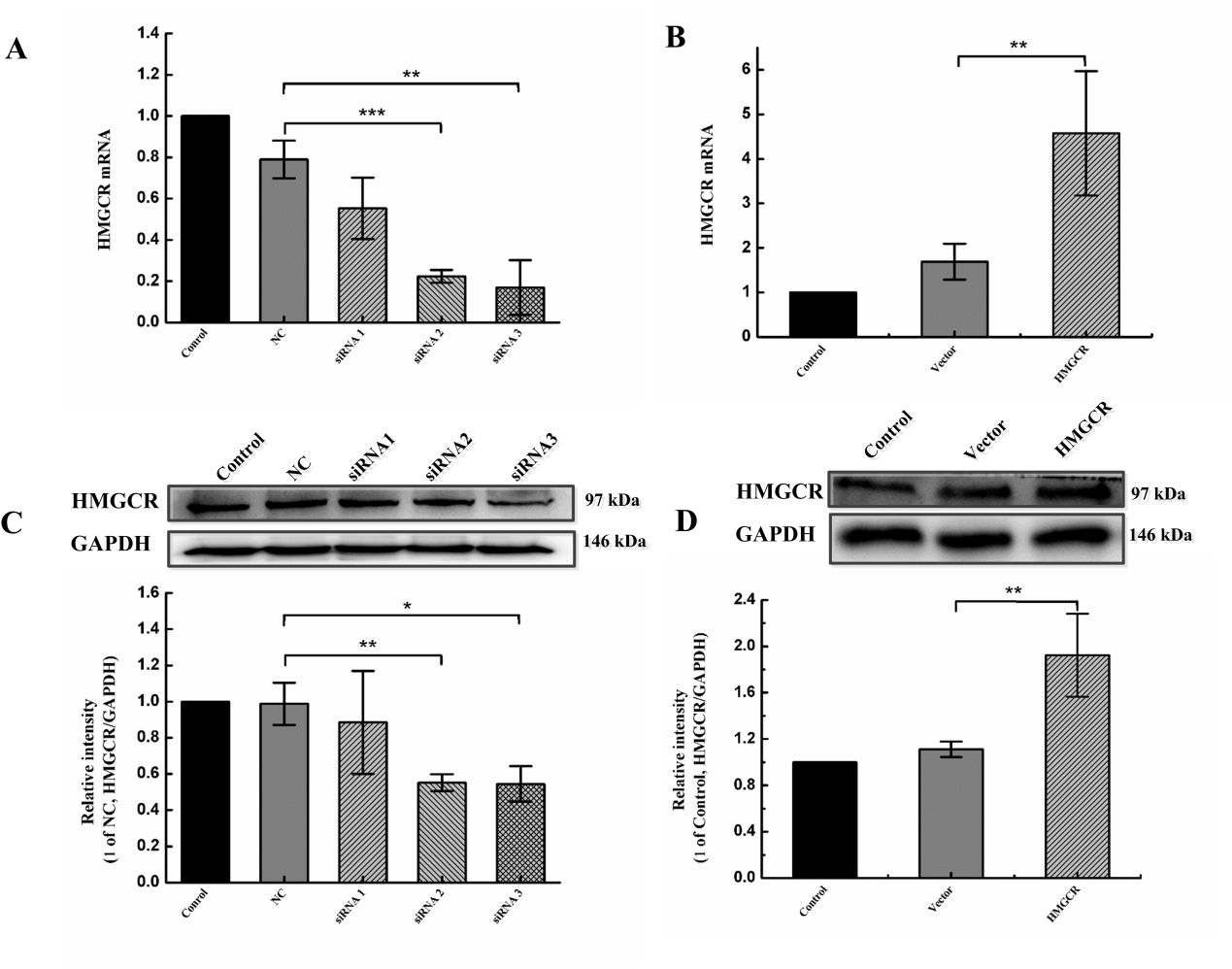


**Supplementary Figure 2.** Transfection effects of different siRNA sequences and overexpressed plasmid in HEK-293T cells. (A) mRNA level of HMGCR after interfering by different siRNA. (B) mRNA level of HMGCR after overexpressing by plasmid. (C) Protein expression of HMGCR after interfering by different siRNA. (D) Protein expression of HMGCR after overexpressing by plasmid. The results are represented as means ± SD from experiments in triplicate. **P <* 0.05, ***P <* 0.05, *** *P <* 0.001, *vs* NC or vector group.

**Supplementary Table 1.** DEPs that expressed in damage group *vs* control group.

| **Protein accession** | **Protein description** | **C/S Ratio** | **Regulated Type** | **C/S P value** | **Gene name** | **Subcellular localization** |
| --- | --- | --- | --- | --- | --- | --- |
| Q06547 | GA-binding protein subunit beta-1 OS=Homo sapiens OX=9606 GN=GABPB1 | 1.242 | Up | 0.039278 | GABPB1 | cytoskeleton |
| P04035 | 3-hydroxy-3-methylglutaryl-coenzyme A reductase OS=Homo sapiens OX=9606 GN=HMGCR | 1.545 | Up | 0.025243 | HMGCR | plasma membrane |
| Q9P2E5 | Chondroitin sulfate glucuronyltransferase OS=Homo sapiens OX=9606 GN=CHPF2 | 1.244 | Up | 0.040377 | CHPF2 | endoplasmic reticulum |
| Q86Y39 | NADH dehydrogenase [ubiquinone] 1 alpha subcomplex subunit 11 OS=Homo sapiens OX=9606 GN=NDUFA11 | 1.253 | Up | 0.0054784 | NDUFA11 | mitochondria |
| Q12982 | BCL2/adenovirus E1B 19 kDa protein-interacting protein 2 OS=Homo sapiens OX=9606 GN=BNIP2 | 1.261 | Up | 0.0128428 | BNIP2 | cytoplasm |
| Q12770 | Sterol regulatory element-binding protein cleavage-activating protein OS=Homo sapiens OX=9606 GN=SCAP | 1.292 | Up | 0.0044155 | SCAP | plasma membrane |
| P05412 | Transcription factor AP-1 OS=Homo sapiens OX=9606 GN=JUN | 1.23 | Up | 0.0162185 | JUN | nucleus |
| Q9H1E3 | Nuclear ubiquitous casein and cyclin-dependent kinase substrate 1 OS=Homo sapiens OX=9606 GN=NUCKS1 | 1.207 | Up | 0.00117963 | NUCKS1 | nucleus |
| Q96S66 | Chloride channel CLIC-like protein 1 OS=Homo sapiens OX=9606 GN=CLCC1 | 1.209 | Up | 0.028641 | CLCC1 | plasma membrane |
| P02794 | Ferritin heavy chain OS=Homo sapiens OX=9606 GN=FTH1 | 1.215 | Up | 0.0035643 | FTH1 | cytoplasm |
| Q9GZV5 | WW domain-containing transcription regulator protein 1 OS=Homo sapiens OX=9606 GN=WWTR1 | 1.39 | Up | 0.0029354 | WWTR1 | nucleus |
| Q13501 | Sequestosome-1 OS=Homo sapiens OX=9606 GN=SQSTM1 | 1.227 | Up | 0.0197988 | SQSTM1 | nucleus |
| Q9NQZ5 | "StAR-related lipid transfer protein 7, mitochondrial OS=Homo sapiens OX=9606 GN=STARD7" | 1.234 | Up | 0.008042 | STARD7 | mitochondria |
| Q9BUB5 | MAP kinase-interacting serine/threonine-protein kinase 1 OS=Homo sapiens OX=9606 GN=MKNK1 | 1.275 | Up | 0.03032 | MKNK1 | nucleus |
| Q16799 | Reticulon-1 OS=Homo sapiens OX=9606 GN=RTN1 | 1.42 | Up | 0.040117 | RTN1 | nucleus |
| Q9H3K2 | Growth hormone-inducible transmembrane protein OS=Homo sapiens OX=9606 GN=GHITM | 0.743 | Down | 0.00161683 | GHITM | plasma membrane |
| O15121 | Sphingolipid delta(4)-desaturase DES1 OS=Homo sapiens OX=9606 GN=DEGS1 | 0.83 | Down | 0.0026221 | DEGS1 | plasma membrane |
| Q02880 | DNA topoisomerase 2-beta OS=Homo sapiens OX=9606 GN=TOP2B | 0.829 | Down | 1.75628E-05 | TOP2B | nucleus |
| Q3KRA9 | Alpha-ketoglutarate-dependent dioxygenase alkB homolog 6 OS=Homo sapiens OX=9606 GN=ALKBH6 | 0.829 | Down | 0.006984 | ALKBH6 | cytoplasm |
| Q96E29 | "Transcription termination factor 3, mitochondrial OS=Homo sapiens OX=9606 GN=MTERF3" | 0.774 | Down | 0.00064312 | MTERF3 | mitochondria |
| O75616 | "GTPase Era, mitochondrial OS=Homo sapiens OX=9606 GN=ERAL1" | 0.821 | Down | 4.0099E-06 | ERAL1 | mitochondria |
| P11388 | DNA topoisomerase 2-alpha OS=Homo sapiens OX=9606 GN=TOP2A | 0.781 | Down | 0.00127827 | TOP2A | nucleus |
| Q15004 | PCNA-associated factor OS=Homo sapiens OX=9606 GN=PCLAF | 0.749 | Down | 0.00114223 | PCLAF | mitochondria |
| Q8WUK0 | Phosphatidylglycerophosphatase and protein-tyrosine phosphatase 1 OS=Homo sapiens OX=9606 GN=PTPMT1 | 0.799 | Down | 0.0041779 | PTPMT1 | mitochondria |
| Q96TA2 | ATP-dependent zinc metalloprotease YME1L1 OS=Homo sapiens OX=9606 GN=YME1L1 | 0.83 | Down | 0.000043737 | YME1L1 | plasma membrane |
| Q8NFI3 | Cytosolic endo-beta-N-acetylglucosaminidase OS=Homo sapiens OX=9606 GN=ENGASE | 0.822 | Down | 0.000101007 | ENGASE | mitochondria |
| Q9NX00 | Transmembrane protein 160 OS=Homo sapiens OX=9606 GN=TMEM160 | 0.804 | Down | 0.03002 | TMEM160 | mitochondria |
| O43548 | Protein-glutamine gamma-glutamyltransferase 5 OS=Homo sapiens OX=9606 GN=TGM5 | 0.652 | Down | 0.0166048 | TGM5 | cytoplasm |
| Q9NPF0 | CD320 antigen OS=Homo sapiens OX=9606 GN=CD320 | 0.829 | Down | 0.0198014 | CD320 | extracellular |
| Q8NGC4 | Olfactory receptor 10G3 OS=Homo sapiens OX=9606 GN=OR10G3 | 0.743 | Down | 0.000097488 | OR10G3 | plasma membrane |
| Q15014 | Mortality factor 4-like protein 2 OS=Homo sapiens OX=9606 GN=MORF4L2 | 0.817 | Down | 0.00020316 | MORF4L2 | nucleus |
| O95571 | "Persulfide dioxygenase ETHE1, mitochondrial OS=Homo sapiens OX=9606 GN=ETHE1" | 0.818 | Down | 0.0101997 | ETHE1 | mitochondria |
| Q5JPI3 | Uncharacterized protein C3orf38 OS=Homo sapiens OX=9606 GN=C3orf38 | 0.756 | Down | 0.000177576 | C3orf38 | cytoplasm |
| Q9NRZ9 | Lymphoid-specific helicase OS=Homo sapiens OX=9606 GN=HELLS | 0.823 | Down | 1.60027E-05 | HELLS | cytoplasm |
| Q9BT30 | "Alpha-ketoglutarate-dependent dioxygenase alkB homolog 7, mitochondrial OS=Homo sapiens OX=9606 GN=ALKBH7" | 0.781 | Down | 0.0079202 | ALKBH7 | mitochondria |
| Q9UBU8 | Mortality factor 4-like protein 1 OS=Homo sapiens OX=9606 GN=MORF4L1 | 0.763 | Down | 0.000123462 | MORF4L1 | nucleus |
| Q9BV57 | "1,2-dihydroxy-3-keto-5-methylthiopentene dioxygenase OS=Homo sapiens OX=9606 GN=ADI1" | 0.449 | Down | 0.00179556 | ADI1 | cytoplasm |
| Q96SZ6 | CDK5 regulatory subunit-associated protein 1 OS=Homo sapiens OX=9606 GN=CDK5RAP1 | 0.826 | Down | 0.034863 | CDK5RAP1 | mitochondria |
| Q96SZ5 | 2-aminoethanethiol dioxygenase OS=Homo sapiens OX=9606 GN=ADO | 0.802 | Down | 0.000083176 | ADO | mitochondria |
| O95677 | Eyes absent homolog 4 OS=Homo sapiens OX=9606 GN=EYA4 | 0.83 | Down | 0.0045819 | EYA4 | cyto_nucl |
| P13498 | Cytochrome b-245 light chain OS=Homo sapiens OX=9606 GN=CYBA | 0.757 | Down | 0.00033571 | CYBA | plasma membrane |
| Q13907 | Isopentenyl-diphosphate Delta-isomerase 1 OS=Homo sapiens OX=9606 GN=IDI1 | 0.684 | Down | 0.000157688 | IDI1 | cytoplasm |
| Q04941 | Proteolipid protein 2 OS=Homo sapiens OX=9606 GN=PLP2 | 0.832 | Down | 0.025178 | PLP2 | plasma membrane |
| Q6P2S7 | Putative tetratricopeptide repeat protein 41 OS=Homo sapiens OX=9606 GN=TTC41P | 0.827 | Down | 0.0183751 | TTC41P | nucleus |
| Q71DI3 | Histone H3.2 OS=Homo sapiens OX=9606 GN=HIST2H3A | 0.793 | Down | 0.0189589 | HIST2H3A | nucleus |
| Q15800 | Methylsterol monooxygenase 1 OS=Homo sapiens OX=9606 GN=MSMO1 | 0.803 | Down | 0.00069832 | MSMO1 | extracellular |
| P49588 | "Alanine--tRNA ligase, cytoplasmic OS=Homo sapiens OX=9606 GN=AARS" | 0.691 | Down | 4.6396E-10 | AARS | cytoplasm |
| Q92896 | Golgi apparatus protein 1 OS=Homo sapiens OX=9606 GN=GLG1 | 0.675 | Down | 0.00022362 | GLG1 | peroxisome |
| Q9UHQ1 | Nuclear prelamin A recognition factor OS=Homo sapiens OX=9606 GN=NARF | 0.817 | Down | 0.0044826 | NARF | cytoplasm |
| O00767 | Acyl-CoA desaturase OS=Homo sapiens OX=9606 GN=SCD | 0.697 | Down | 0.00045847 | SCD | cytoplasm |
| Q9HAW4 | Claspin OS=Homo sapiens OX=9606 GN=CLSPN | 0.726 | Down | 0.00076076 | CLSPN | nucleus |
| Q8IZT6 | Abnormal spindle-like microcephaly-associated protein OS=Homo sapiens OX=9606 GN=ASPM | 0.82 | Down | 0.0166449 | ASPM | nucleus |
| P00374 | Dihydrofolate reductase OS=Homo sapiens OX=9606 GN=DHFR | 0.825 | Down | 0.000021575 | DHFR | cytoplasm |
| Q14527 | Helicase-like transcription factor OS=Homo sapiens OX=9606 GN=HLTF | 0.804 | Down | 0.0026228 | HLTF | nucleus |
| Q9Y4G8 | Rap guanine nucleotide exchange factor 2 OS=Homo sapiens OX=9606 GN=RAPGEF2 | 0.758 | Down | 0.000042864 | RAPGEF2 | cytoplasm |
| Q9BZL1 | Ubiquitin-like protein 5 OS=Homo sapiens OX=9606 GN=UBL5 | 0.781 | Down | 8.2007E-07 | UBL5 | extracellular |
| P14543 | Nidogen-1 OS=Homo sapiens OX=9606 GN=NID1 | 0.821 | Down | 0.029823 | NID1 | extracellular |
| P01034 | Cystatin-C OS=Homo sapiens OX=9606 GN=CST3 | 0.792 | Down | 0.0046209 | CST3 | extracellular |
| Q8NEZ2 | Vacuolar protein sorting-associated protein 37A OS=Homo sapiens OX=9606 GN=VPS37A | 0.79 | Down | 0.049865 | VPS37A | mitochondria |
| Q5R3I4 | Tetratricopeptide repeat protein 38 OS=Homo sapiens OX=9606 GN=TTC38 | 0.789 | Down | 0.00031946 | TTC38 | cytoplasm |
| P22830 | "Ferrochelatase, mitochondrial OS=Homo sapiens OX=9606 GN=FECH" | 0.798 | Down | 1.8686E-06 | FECH | mitochondria |
| Q8N5C6 | S1 RNA-binding domain-containing protein 1 OS=Homo sapiens OX=9606 GN=SRBD1 | 0.817 | Down | 0.035837 | SRBD1 | nucleus |
| Q7Z4H3 | HD domain-containing protein 2 OS=Homo sapiens OX=9606 GN=HDDC2 | 0.68 | Down | 0.0020964 | HDDC2 | mitochondria |
| Q9HB07 | "UPF0160 protein MYG1, mitochondrial OS=Homo sapiens OX=9606 GN=C12orf10" | 0.798 | Down | 0.000040497 | C12orf10 | mitochondria |
| Q8WVX3 | Uncharacterized protein C4orf3 OS=Homo sapiens OX=9606 GN=C4orf3 | 0.819 | Down | 0.00013689 | C4orf3 | plasma membrane |
| Q5JTZ9 | "Alanine--tRNA ligase, mitochondrial OS=Homo sapiens OX=9606 GN=AARS2" | 0.806 | Down | 0.000077598 | AARS2 | mitochondria |
| P34949 | Mannose-6-phosphate isomerase OS=Homo sapiens OX=9606 GN=MPI | 0.824 | Down | 0.000119783 | MPI | mitochondria |
| Q9UK39 | Nocturnin OS=Homo sapiens OX=9606 GN=NOCT | 0.749 | Down | 0.039683 | NOCT | mitochondria |
| Q15327 | Ankyrin repeat domain-containing protein 1 OS=Homo sapiens OX=9606 GN=ANKRD1 | 0.74 | Down | 0.00038336 | ANKRD1 | cyto_nucl |
| Q9BW92 | "Threonine--tRNA ligase, mitochondrial OS=Homo sapiens OX=9606 GN=TARS2" | 0.654 | Down | 0.000103953 | TARS2 | mitochondria |

**Supplementary Table 2.** DEPs that expressed in C3G intervention group *vs* damage group.

| **Protein accession** | **Protein description** | **C/S Ratio** | **Regulated Type** | **C/S P value** | **Gene name** | **Subcellular localization** |
| --- | --- | --- | --- | --- | --- | --- |
| Q9UK39 | Nocturnin OS=Homo sapiens OX=9606 GN=NOCT | 1.346 | Up | 0.038918 | NOCT | mitochondria |
| Q7Z7K0 | COX assembly mitochondrial protein homolog OS=Homo sapiens OX=9606 GN=CMC1 | 1.224 | Up | 0.00047983 | CMC1 | extracellular |
| Q15327 | Ankyrin repeat domain-containing protein 1 OS=Homo sapiens OX=9606 GN=ANKRD1 | 1.292 | Up | 0.00058135 | ANKRD1 | cyto_nucl |
| Q02880 | DNA topoisomerase 2-beta OS=Homo sapiens OX=9606 GN=TOP2B | 1.241 | Up | 0.000003876 | TOP2B | nucleus |
| O14734 | Acyl-coenzyme A thioesterase 8 OS=Homo sapiens OX=9606 GN=ACOT8 | 1.258 | Up | 0.044977 | ACOT8 | cytoplasm |
| Q5JPI3 | Uncharacterized protein C3orf38 OS=Homo sapiens OX=9606 GN=C3orf38 | 1.276 | Up | 0.000104617 | C3orf38 | cytoplasm |
| P32929 | Cystathionine gamma-lyase OS=Homo sapiens OX=9606 GN=CTH | 1.26 | Up | 0.024555 | CTH | cytoplasm |
| Q04941 | Proteolipid protein 2 OS=Homo sapiens OX=9606 GN=PLP2 | 1.204 | Up | 0.0181433 | PLP2 | plasma membrane |
| Q6P2S7 | Putative tetratricopeptide repeat protein 41 OS=Homo sapiens OX=9606 GN=TTC41P | 1.246 | Up | 0.0099006 | TTC41P | nucleus |
| Q9Y4G8 | Rap guanine nucleotide exchange factor 2 OS=Homo sapiens OX=9606 GN=RAPGEF2 | 1.4 | Up | 0.000058483 | RAPGEF2 | cytoplasm |
| P46013 | Proliferation marker protein Ki-67 OS=Homo sapiens OX=9606 GN=MKI67 | 1.246 | Up | 0.000102217 | MKI67 | mitochondria |
| P11388 | DNA topoisomerase 2-alpha OS=Homo sapiens OX=9606 GN=TOP2A | 1.262 | Up | 0.00002045 | TOP2A | nucleus |
| P07949 | Proto-oncogene tyrosine-protein kinase receptor Ret OS=Homo sapiens OX=9606 GN=RET | 0.766 | Down | 0.0114235 | RET | plasma membrane |
| Q9Y548 | Protein YIPF1 OS=Homo sapiens OX=9606 GN=YIPF1 | 0.822 | Down | 0.00027539 | YIPF1 | plasma membrane |
| Q8IWD4 | Coiled-coil domain-containing protein 117 OS=Homo sapiens OX=9606 GN=CCDC117 | 0.801 | Down | 0.0110615 | CCDC117 | nucleus |
| A6NEE1 | Pleckstrin homology domain-containing family D member 1 OS=Homo sapiens OX=9606 GN=PLEKHD1 | 0.799 | Down | 0.00086255 | PLEKHD1 | nucleus |
| P04035 | 3-hydroxy-3-methylglutaryl-coenzyme A reductase OS=Homo sapiens OX=9606 GN=HMGCR | 0.735 | Down | 0.039765 | HMGCR | plasma membrane |
| Q8WXD5 | Gem-associated protein 6 OS=Homo sapiens OX=9606 GN=GEMIN6 | 0.795 | Down | 0.041236 | GEMIN6 | cytoplasm |
| Q9Y5G2 | Protocadherin gamma-B2 OS=Homo sapiens OX=9606 GN=PCDHGB2 | 0.747 | Down | 0.000159698 | PCDHGB2 | endoplasmic reticulum |
| Q5T3J3 | Ligand-dependent nuclear receptor-interacting factor 1 OS=Homo sapiens OX=9606 GN=LRIF1 | 0.823 | Down | 0.0135834 | LRIF1 | nucleus |
| Q12770 | Sterol regulatory element-binding protein cleavage-activating protein OS=Homo sapiens OX=9606 GN=SCAP | 0.588 | Down | 0.0031398 | SCAP | plasma membrane |
| Q9Y6V0 | Protein piccolo OS=Homo sapiens OX=9606 GN=PCLO | 0.78 | Down | 3.0474E-06 | PCLO | cyto_nucl |
| O94885 | SAM and SH3 domain-containing protein 1 OS=Homo sapiens OX=9606 GN=SASH1 | 0.489 | Down | 0.0005227 | SASH1 | nucleus |
| P04179 | "Superoxide dismutase [Mn], mitochondrial OS=Homo sapiens OX=9606 GN=SOD2" | 0.831 | Down | 0.00022004 | SOD2 | mitochondria |
| Q9BUB5 | MAP kinase-interacting serine/threonine-protein kinase 1 OS=Homo sapiens OX=9606 GN=MKNK1 | 0.83 | Down | 0.006275 | MKNK1 | nucleus |
| P51688 | N-sulphoglucosamine sulphohydrolase OS=Homo sapiens OX=9606 GN=SGSH | 0.778 | Down | 0.04766 | SGSH | extracellular |
| Q9GZV5 | WW domain-containing transcription regulator protein 1 OS=Homo sapiens OX=9606 GN=WWTR1 | 0.786 | Down | 0.043736 | WWTR1 | nucleus |
| Q13530 | Serine incorporator 3 OS=Homo sapiens OX=9606 GN=SERINC3 | 0.683 | Down | 0.027856 | SERINC3 | plasma membrane |
